# Two-year follow-up of KTE-X19 in patients with relapsed or refractory adult B-cell acute lymphoblastic leukemia in ZUMA-3 and its contextualization with SCHOLAR-3, an external historical control study

**DOI:** 10.1186/s13045-022-01379-0

**Published:** 2022-12-10

**Authors:** Bijal D. Shah, Armin Ghobadi, Olalekan O. Oluwole, Aaron C. Logan, Nicolas Boissel, Ryan D. Cassaday, Thibaut Leguay, Michael R. Bishop, Max S. Topp, Dimitrios Tzachanis, Kristen M. O’Dwyer, Martha L. Arellano, Yi Lin, Maria R. Baer, Gary J. Schiller, Jae H. Park, Marion Subklewe, Mehrdad Abedi, Monique C. Minnema, William G. Wierda, Daniel J. DeAngelo, Patrick Stiff, Deepa Jeyakumar, Jinghui Dong, Sabina Adhikary, Lang Zhou, Petra C. Schuberth, Imi Faghmous, Behzad Kharabi Masouleh, Roch Houot

**Affiliations:** 1 Moffitt Cancer Center, Tampa, FL 33612 USA; 2 Washington University School of Medicine, St Louis, MO USA; 3 Vanderbilt University Cancer Center, Nashville, TN USA; 4 UCSF Medical Center, San Francisco, CA USA; 5 Hôpital Saint-Louis, Paris, France; 6 University of Washington, Fred Hutchinson Cancer Center, Seattle, WA USA; 7 Service d’hématologie Clinique Et Thérapie Cellulaire, Hopital du Haut-Leveque CHU de Bordeaux, Bordeaux, France; 8 The University of Chicago Medicine, Chicago, IL USA; 9 Medizinische Klinik Und Poliklinik II, Universitätsklinikum Würzburg, Würzburg, Germany; 10 University of California San Diego, San Diego, CA USA; 11 Wilmot Cancer Institute of University of Rochester, Rochester, NY USA; 12 Winship Cancer Institute of Emory University, Atlanta, GA USA; 13 Mayo Clinic, Rochester, MN USA; 14 University of Maryland Marlene and Stewart Greenebaum Comprehensive Cancer Center, Baltimore, MD USA; 15 David Geffen School of Medicine at UCLA, Los Angeles, CA USA; 16 Memorial Sloan Kettering Cancer Center, New York, NY USA; 17 Ludwig-Maximilians-Universität München, Munich, Germany; 18 University of California Davis Comprehensive Cancer Center, Sacramento, CA USA; 19 University Medical Center Utrecht (on behalf of HOVON/LLPC), Utrecht, The Netherlands; 20 The University of Texas MD Anderson Cancer Center, Houston, TX USA; 21 Dana-Farber Cancer Institute, Boston, MA USA; 22 Loyola University Chicago Stritch School of Medicine, Maywood, IL USA; 23 University of California Irvine Medical Center, Orange, CA USA; 24 Kite, a Gilead Company, Santa Monica, CA USA; 25 CHU Rennes, Univ Rennes, Inserm & EFS, Rennes, France

**Keywords:** B-precursor acute lymphoblastic leukemia, Brexucabtagene autoleucel, CAR T-cell therapy, KTE-X19, SCHOLAR-3, ZUMA-3

## Abstract

**Background:**

Brexucabtagene autoleucel (KTE-X19) is an autologous anti-CD19 CAR T-cell therapy approved in the USA to treat adult patients with relapsed or refractory B-precursor acute lymphoblastic leukemia (R/R B-ALL) based on ZUMA-3 study results. We report updated ZUMA-3 outcomes with longer follow-up and an extended data set along with contextualization of outcomes to historical standard of care.

**Methods:**

Adults with R/R B-ALL received a single infusion of KTE-X19 (1 × 10^6^ CAR T cells/kg). Long-term post hoc subgroup assessments of ZUMA-3 were conducted. Outcomes from matched patients between historical clinical trials and ZUMA-3 patients were assessed in the retrospective historical control study SCHOLAR-3.

**Results:**

After 26.8-months median follow-up, the overall complete remission (CR) rate (CR + CR with incomplete hematological recovery) among treated patients (*N* = 55) in phase 2 was 71% (56% CR rate); medians for duration of remission and overall survival (OS) were 14.6 and 25.4 months, respectively. Most patients responded to KTE-X19 regardless of age or baseline bone marrow blast percentage, but less so in patients with > 75% blasts. No new safety signals were observed. Similar outcomes were observed in a pooled analysis of phase 1 and 2 patients (*N* = 78). In SCHOLAR-3, the median OS for treated patients from ZUMA-3 (*N* = 49) and matched historical controls (*N* = 40) was 25.4 and 5.5 months, respectively.

**Conclusions:**

These data, representing the longest follow-up of CAR T-cell therapy in a multicenter study of adult R/R B-ALL, suggest that KTE-X19 provides a clinically meaningful survival benefit with manageable toxicity in this population.

*Trial Registration*: NCT02614066.

**Supplementary Information:**

The online version contains supplementary material available at 10.1186/s13045-022-01379-0.

## Background

Adults with relapsed or refractory B-precursor acute -lymphoblastic leukemia (R/R B-ALL) face an overall poor prognosis despite the availability of newer treatment options such as blinatumomab and inotuzumab ozogamicin, which result in median overall survival (OS) of < 8 months [1–3]. Improvements in survival rates have largely been limited to younger patients, with only 34% of patients with B-ALL 40–64 years of age living 5 years after diagnosis, whereas 88% of patients with B-ALL < 20 years of age are living 5 years after diagnosis [4]. In addition, several high-risk disease characteristics, such as high disease burden at diagnosis, are considered poor prognostic indicators of response to salvage therapy and survival [5], highlighting an unmet need for more effective therapies in these patient populations. Although outcomes in adult patients with R/R B-ALL are generally considered to be poor with current standard-of-care regimens, studies aimed at assessing outcomes across historical clinical trials that accurately estimate the unmet need are limited.

Brexucabtagene autoleucel (KTE-X19) is an autologous anti-CD19 chimeric antigen receptor (CAR) T-cell therapy that was approved in the USA to treat adults with R/R B-ALL based on the positive results of the ZUMA-3 study [6]. After 16.4-months median follow-up in phase 2 of ZUMA-3, KTE-X19 demonstrated compelling clinical efficacy and manageable safety in a heavily pretreated population, with an overall complete remission (CR) rate (CR + CR with incomplete hematological recovery [CRi]) of 71% (95% CI 57–82; *N* = 55). The median overall survival (OS) was 18.2 months (95% CI 15.9–not estimable [NE]) in all treated patients and not reached (NR) among responders [7].

To better assess the unmet need for patients with R/R B-ALL and to estimate the benefit of KTE-X19 compared with standard-of-care regimens in this setting, we recently performed the retrospective, external historical control study SCHOLAR-3 [8]. Outcome comparisons were made based on matched individual patient-level data from historical clinical trials with ZUMA-3 patients [8]. SCHOLAR-3 results demonstrated high unmet need among patients with R/R B-ALL with standard-of-care therapies providing a median OS of 5.5 months (*n* = 40) in historical clinical trials, whereas in matched patients receiving KTE-X19 therapy in ZUMA-3, the median OS was 18.2 months (*n* = 49) [8].

Here we report updated efficacy, safety, and pharmacology data of KTE-X19 with longer follow-up in phase 2 ZUMA-3 patients (*N* = 55) and a newly conducted pooled analysis by independent central review of phase 1 and 2 patients (*N* = 78) who received the pivotal dose (1 × 10^6^ CAR T cells/kg). Post hoc analyses of subgroups by age and baseline bone marrow blast percentages are also reported. In addition, updated results of the SCHOLAR-3 external historical control study with extended follow-up are reported to contextualize the updated ZUMA-3 findings in the current R/R B-ALL treatment paradigm.

## Methods

### ZUMA-3

#### Study design and patients

Detailed study procedures for the single-arm, multicenter, registrational, phase 1/2 ZUMA-3 study (NCT02614066) have been reported (Additional file 1: Supplemental Methods) [7, 9]. Briefly, adult patients (≥ 18 years) had R/R B-ALL with morphological disease in the bone marrow (> 5% blasts). Previous blinatumomab and/or allogeneic stem cell transplant (alloSCT) were permitted. The study was conducted in accordance with the principles of the Declaration of Helsinki, the institutional review board or independent ethics committee at each study site approved the protocol, and all patients provided written informed consent.

#### Procedures

Patients in ZUMA-3 underwent leukapheresis followed by conditioning chemotherapy (intravenous fludarabine 25 mg/m^2^ on days - 4, -3, and -2; and intravenous cyclophosphamide 900 mg/m^2^ on day -2) and a single intravenous infusion of 1 × 10^6^ CAR T cells/kg on day 0. Bridging therapy was allowed per physician’s discretion as previously reported and outlined in the protocol (Additional file 1: Supplemental Methods) [7]. Following bridging therapy, bone marrow blast levels were reevaluated by day -4 preinfusion. AlloSCT, administered at investigator’s discretion, was allowed as subsequent consolidative therapy following KTE-X19, but was not protocol defined. Patients were eligible to receive a second infusion of KTE-X19 in limited circumstances (Additional file 1: Supplemental Methods).

#### Outcomes

The primary endpoint in ZUMA-3 was the overall CR/CRi rate by indepent central review. Key secondary endpoints included duration of remission (DOR) and relapse-free survival (RFS) with patients undergoing new anticancer therapies (including alloSCT) censored (Additional file 1: Supplemental Methods); OS; alloSCT rate; and safety. Exploratory endpoints included CD19 positive CAR T cell levels and B cell levels in blood.

Post hoc efficacy assessments of subgroups were performed by age (18–25, 18–39, 40–59, ≥ 60 years) and baseline bone marrow blast percentages (0–5%, > 5–25%, > 25–50%, > 50–75%, > 75–100%) following bridging therapy and prior to KTE-X19 infusion.

#### Statistical analyses

Updated efficacy, safety, and translational endpoints in ZUMA-3 are reported in phase 2 treated patients and in a combined analysis of phase 1 and 2 patients treated at the pivotal dose of KTE-X19 (1 × 10^6^ CAR T cells/kg). The analyzed data sets are described in Additional file 1: Supplemental Methods.

Time-to-event endpoints were analyzed using the Kaplan–Meier method, and subgroup analyses were descriptive. Additional statistical analysis details are provided in Additional file 1: Supplemental Methods.

### SCHOLAR-3 analysis

Detailed methodology of the retrospective cohort study SCHOLAR-3 was previously reported (Additional file 1: Supplemental Methods) [8]. Briefly, propensity scoring was used to match adult patients with R/R B‑ALL treated in historical clinical trials (synthetic control arm [SCA]) with ZUMA-3-treated patients based on key baseline characteristics and prior therapies. The study consisted of three patient-matched historical control cohorts, as follows: (1) SCA-1: patients who were previously naïve to blinatumomab and inotuzumab at the time of enrollment in historical trials (timepoint of inclusion in the SCHOLAR-3 analysis) in which they may have received blinatumomab or inotuzumab; (2) SCA-2: patients who were previously treated with blinatumomab or inotuzumab at the time of enrollment in historical trials (timepoint of inclusion in SCHOLAR-3 analysis) in which they may have received blinatumomab or inotuzumab; and (3) SCA-combined: SCA-1 and SCA-2 combined data set (Additional file 1: Supplemental Methods) [8]. These cohorts were compared with matched ZUMA-3 patients who were previously naïve to blinatumomab and inotuzumab, previously treated with blinatumomab or inotuzumab, and any pretreatment status, respectively. The primary endpoint was the CR/CRi rate of the SCA-1 cohort analysis. Secondary endpoints included alloSCT and RFS rates in cohort SCA-1, and OS in all three cohort analyses. Efficacy outcomes in both intention-to-treat (ITT) and treated patients in ZUMA-3 were compared with SCHOLAR-3 cohorts.

#### Role of the funding source

The study sponsor, in collaboration with the authors, participated in the study design; the collection, analysis, and interpretation of data; and writing of the report.

## Results

### ZUMA-3 patients

As previously reported, 71 patients were enrolled and leukapheresed in phase 2, and 55 patients received KTE-X19 (reasons for discontinuing prior to infusion were previously reported) [7]. As of July 23, 2021, median potential follow-up time was 26.8 months (range, 20.7–32.6). Most patients were heavily pretreated, as previously reported [7]. Baseline characteristics by age and baseline bone marrow blast percentage subgroups are described in the Supplemental Results and Table S1.

In the larger pooled analysis of phase 1 (*n* = 23) and phase 2 patients (*n* = 55) who received the pivotal dose of KTE-X19, the median potential follow-up time was 29.7 months (range, 20.7–58.3). Baseline characteristics for all patients (*N* = 78) and by subgroups were consistent with those in phase 2 treated patients (Additional file 1: Supplementary Results and Table S2).

### Updated phase 2 outcomes

For treated phase 2 patients (*N* = 55), the CR/CRi rate per independent central review remained 71% (95% CI 57–82; 56% CR rate) since the primary analysis (Table 1; MRD-negativity previously reported) [7]. Eleven patients (20%; eight CR, two CRi, one blast-free hypoplastic or aplastic bone marrow [BFBM]) proceeded to subsequent alloSCT after KTE-X19 treatment (without additional anti-cancer therapy prior to receiving alloSCT), including one additional patient (CR) since the primary analysis. Median time to alloSCT was 101 days (range, 60–390) after KTE-X19 infusion.

**Table 1:** Summary of efficacy and durability outcomes in all phase 2 treated patients (*N* = 55) and pooled phase 1 and 2 treated patients (*N* = 78) by age and baseline bone marrow blast percentage

	*N*	Overall CR rate, *n* (%)	CR, *n* (%)	CRi, *n* (%)	BFBM, *n* (%)	No response, *n* (%)	Median DOR, mo. (95% CI)	Median RFS, mo. (95% CI)	Median OS, mo. (95% CI)
Phase 2 treated	55	39 (70.9)	31 (56.4)	8 (14.5)	4 (7.3)	9 (16.4)	14.6 (9.4–NE)	11.6 (2.7–20.5)	25.4 (16.2–NE)
Age, years									
18–25	12	8 (66**.**7)	7 (58**.**3)	1 (8**.**3)	1 (8**.**3)	1 (8**.**3)	16**.**6 (14**.**6–NE)	15**.**5 (0**.**0–NE)	NR (0**.**6–NE)
18–39	26	16 (61**.**5)	14 (53**.**8)	2 (7**.**7)	2 (7**.**7)	5 (19**.**2)	18**.**6 (12**.**8–NE)	14**.**2 (0**.**0–20**.**5)	25**.**4 (9**.**5–NE)
40–59	20	14 (70**.**0)	11 (55**.**0)	3 (15**.**0)	2 (10**.**0)	4 (20**.**0)	10**.**3 (1**.**3–NE)	11**.**6 (0**.**0–NE)	26**.**0 (7**.**6–NE)
≥ 60	9	9 (100)	6 (66**.**7)	3 (33**.**3)	0	0	9**.**4 (1**.**8–NE)	11**.**7 (2**.**8–NE)	NR (12**.**2–NE)
Baseline BM blasts*									
≤ 5%	5	4 (80)	4 (80)	0	1 (20)	0	14**.**4 (1**.**3–NE)	5**.**6 (0**.**0–NE)	NR (8**.**8–NE)
> 5%–25%	10	9 (90)	7 (70)	2 (20)	0	1 (10)	23**.**6 (1**.**8–NE)	25**.**4 (0**.**0–NE)	25**.**4 (8**.**3–NE)
> 25%–50%	11	10 (90.9)	9 (81**.**8)	1 (9**.**1)	0	0	18**.**6 (9**.**4–NE)	20**.**5 (10**.**3–NE)	NR (9**.**0–NE)
> 50%–75%	10	8 (80)	5 (50)	3 (30)	1 (10)	1 (10)	20**.**0 (1**.**0–NE)	6**.**1 (0**.**0–NE)	NR (2**.**1–NE)
> 75%–100%	19	8 (42**.**1)	6 (31**.**6)	2 (10**.**5)	2 (10**.**5)	7 (36**.**8)	9**.**6 (0**.**8–12**.**8)	0**.**0 (0**.**0–11**.**6)	14**.**2 (2**.**2–NE)
Phase 1 and 2	78	57 (73.1)	47 (60.3)	10 (12.8)	6 (7.7)	12 (15.4)	18.6 (9.6–NE)	11.7 (6.1–20.5)	25.4 (16.2–NE)
Age, years									
18–25	15	11 (73**.**3)	9 (60**.**0)	2 (13**.**3)	1 (6**.**7)	1 (6**.**7)	14**.**6 (0**.**7–NE)	15**.**5 (0**.**0–NE)	23**.**2 (9**.**0–NE)
18–39	36	25 (69**.**4)	21 (58**.**3)	4 (11**.**1)	2 (5**.**6)	6 (16**.**7)	18**.**6 (12**.**8–NE)	14**.**2 (2**.**3–NE)	23**.**2 (14**.**2–NE)
40–59	27	19 (70**.**4)	16 (59**.**3)	3 (11**.**1)	3 (11**.**1)	5 (18**.**5)	20.0 (4**.**7–NE)	7**.**7 (0**.**0–22**.**1)	26**.**0 (8**.**3–NE)
≥ 60	15	13 (86**.**7)	10 (66**.**7)	3 (20**.**0)	1 (6**.**7)	1 (6**.**7)	NR (1**.**8–NE)	14**.**4 (2**.**8–NE)	47**.**0 (12**.**2–NE)
Baseline BM blasts*									
0–5%	8	6 (75**.**0)	6 (75**.**0)	0	1 (12**.**5)	1 (12**.**5)	4.9 (1**.**3–NE)	5**.**6 (0**.**0–NE)	26**.**0 (2**.**2–NE)
> 5%–25%	14	12 (85**.**7)	10 (71**.**4)	2 (14**.**3)	1 (7**.**1)	1 (7**.**1)	23**.**6 (1**.**8–NE)	25**.**4 (2**.**8–NE)	25**.**4 (21**.**9–NE)
> 25%–50%	12	10 (83**.**3)	9 (75**.**0)	1 (8**.**3)	1 (8**.**3)	0	18**.**6 (9**.**4–NE)	20**.**5 (0**.**0–NE)	NR (1**.**7–NE)
> 50%–75%	14	12 (85**.**7)	8 (57**.**1)	4 (28**.**6)	1 (7**.**1)	1 (7**.**1)	20**.**0 (5**.**2–NE)	22**.**1 (1**.**8–NE)	NR (3**.**2–NE)
> 75%–100%	30	17 (56**.**7)	14 (46**.**7)	3 (10**.**0)	2 (6**.**7)	9 (30**.**0)	10**.**3 (1**.**3–NE)	2**.**7 (0**.**0–11**.**7)	16**.**1 (9**.**5–NE)

^*^Measured after bridging therapy and prior to KTE-X19 infusion

BFBM = blast-free hypoplastic or aplastic bone marrow. BM = bone marrow. CR = complete remission. CRi = complete remission with incomplete hematological recovery. DOR = duration of remission. mITT = modified intention-to-treat. NE = not estimable. NR = not reached. OS = overall survival. RFS = relapse-free survival

**Fig. 1:**
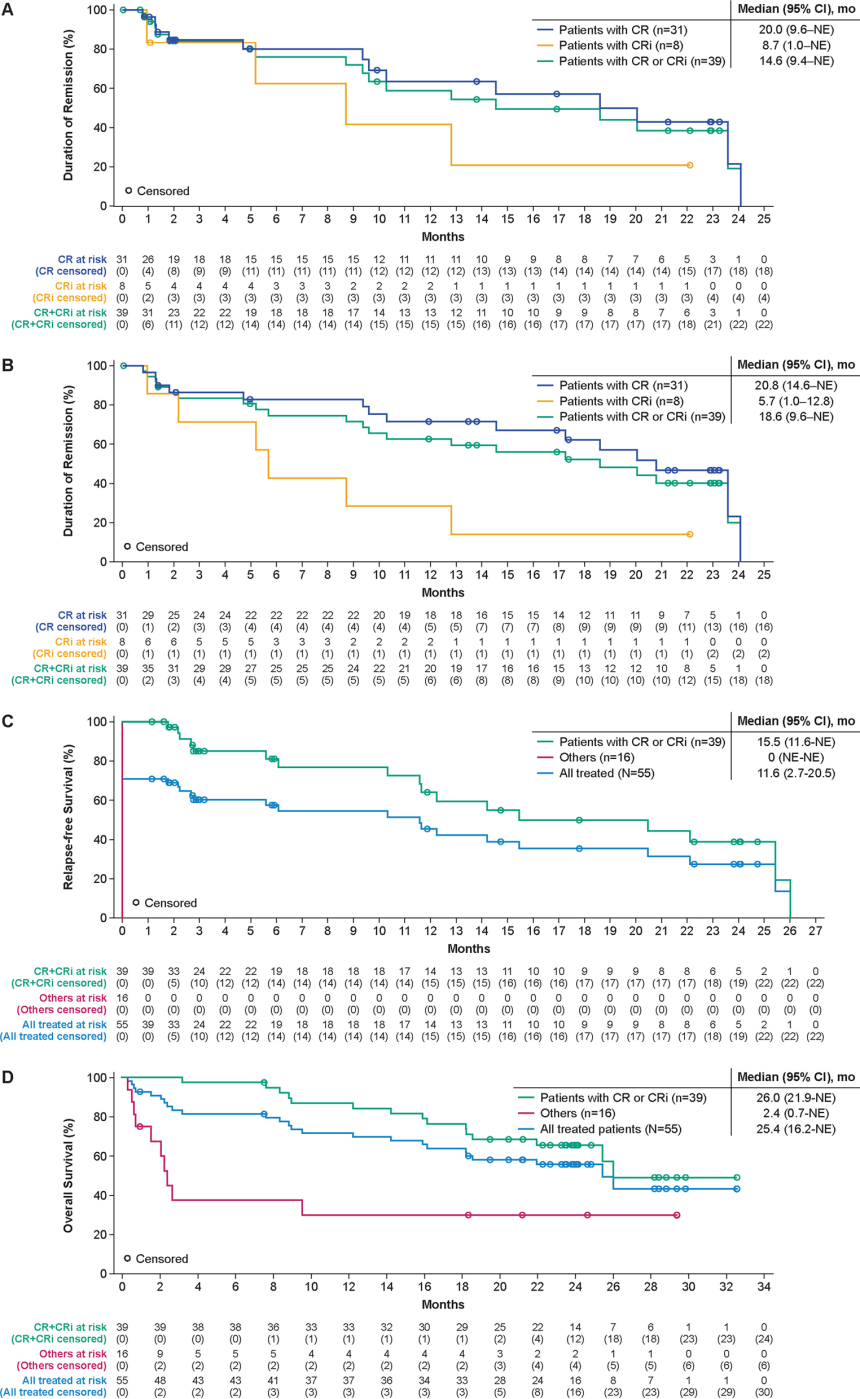
Duration of remission, relapse-free survival, and overall survival for all phase 2 treated patients (*N* = 55). Kaplan–Meier estimates of the duration of remission by central assessment, with (**A**) and without (**B**) censoring of patients at subsequent allogeneic stem cell transplant. **C** Kaplan–Meier estimate of relapse-free survival by central assessment, with censoring of patients at subsequent allogeneic stem cell transplant. **D** Kaplan–Meier estimate of overall survival. CR = complete remission. CRi = complete remission with incomplete hematological recovery. Mo = month. NE = not estimable

**Fig. 2:**
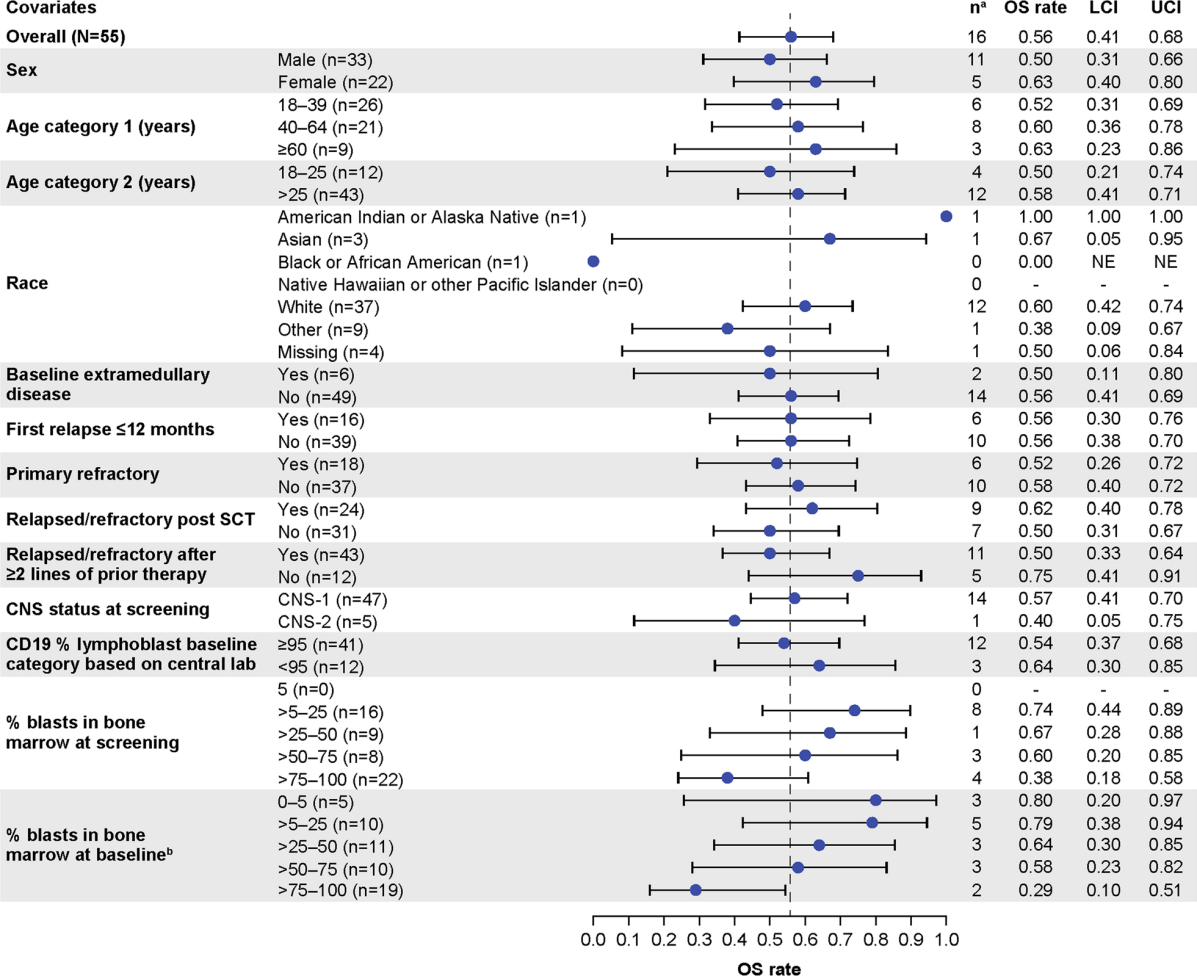
Subgroup analysis of OS at 24 months in phase 2 treated patients (*N* = 55). ^a^Number of subjects at risk at Month 24. ^b^Assessed after bridging therapy and prior to KTE-X19 infusion. CNS = central nervous system. LCI = lower confidence interval. OS = overall survival; SCT = stem cell transplant. UCI = upper confidence interval

Median DOR with and without censoring patients at subsequent alloSCT was 14.6 months (95% CI 9.4–NE); and 18.6 months (95% CI 9.6–NE), respectively (Fig. 1A and B). At data cutoff, 23 (59%) of the 39 patients with CR/CRi were still alive; six (15%) of the 39 were in ongoing remission without additional therapy; ten (26%) proceeded to subsequent alloSCT while in remission (at data cutoff, six remained in remission, one relapsed, and three had died); six (15%) proceeded to other anticancer therapies while in remission (other therapies, Table S3; at data cutoff, four remained in remission and two had died); 14 (36%) relapsed; and three (8%) died. Median RFS censored at subsequent alloSCT was 11.6 months (95% CI 2.7–20.5) for all treated patients (*N* = 55) and 15.5 months (95% CI 11.6–NE) for responders (*n* = 39) (Fig. 1C). Without censoring at subsequent alloSCT, median RFS was 11.7 months (95% CI 2.8–20.5) for all treated patients and 20.5 months (95% CI 11.7–NE) for responders (Additional file 1: Figure S1). Median OS was 25.4 months (95% CI 16.2–NE) for all treated patients, 26.0 months (95% CI 21.9–NE) for responders (Fig. 1D), and not reached for those with CR. OS rates at 24 months were largely similar among prespecified age subgroups but trended lower among patient subgroups with higher bone marrow blasts at baseline (Fig. 2). Two patients, who had disease progression after achieving remission with KTE-X19 (both CD19 positive at relapse), received second KTE-X19 infusions 6.6 and 14 months after the initial infusion, respectively; both had no response as their best response to retreatment.

Among all enrolled phase 2 patients (ITT; *N* = 71), 39 (55%) achieved CR/CRi by independent central review with medians for DOR, RFS, and OS being 14.6 months (95% CI 9.4–NE), 3.7 months (95% CI 0.0–12.9), and 23.1 months (95% CI 10.4–NE), respectively (Table S4).

No new safety signals were observed in ZUMA-3 with longer follow-up. Since the time of the primary analysis, the proportion of patients with grade ≥ 3 adverse events (AEs) was unchanged and no new-onset cytokine release syndrome (CRS), neurological events, infections, secondary malignancies, tumor lysis syndrome, or hypogammaglobulinemia of any grade occurred. One patient had an ongoing neurological event of grade 1 finger numbness; all other CRS and neurological events were resolved by data cutoff. Seven patients (13%) had ongoing cytopenias of any grade on or after day 93 post KTE-X19 infusion; all cytopenias were resolved by data cutoff. One new grade 5 TEAE occurred since the primary analysis: graft vs host disease (GVHD) on day 773 that was deemed not treatment-related by the investigator. In total, 25 (45%) treated patients had died as of the data cutoff date, including five patients who had died in the time between the primary analysis and data cutoff: one due to GVHD as previously described, one due to progressive disease (day 564), and three due to other causes (one due to COVID-19 [day 791] and two after alloSCT [days 554 and 667]). No replication-competent retrovirus cases occurred at any time during the study.

Median time to peak CAR T-cell levels in blood after KTE-X19 infusion in phase 2 was 15 days, with a rapid decline to undetectable levels in most evaluable patients (22/28 [79%]) by month 6 [7], and all evaluable patients (*n* = 10) by month 24 (Additional file 1: Fig. S2). At data cutoff, median peak and area under the curve (AUC) CAR T-cell levels appeared highest in evaluable patients with ongoing remission, trended higher in older patient subgroups (Additional file 1: Fig. S3 and Table S5), and as previously reported, trended lower in patients with higher percentages of baseline bone marrow blasts [7].

### Pooled phase 1 and 2 efficacy outcomes

For the larger pooled analysis of phase 1 and 2 patients (*N* = 78) who received the pivotal dose, the CR/CRi rate by independent central review was 73% (95% CI 62–82), with a 60% CR rate (Table 1), consistent with investigator-assessed rates previously reported with shorter follow-up [7]. Fifteen patients (19%; 10 CR, 3 CRi, 1 BFBM, and 1 partial response) proceeded to subsequent alloSCT after KTE-X19 treatment. Median DOR with and without censoring patients at subsequent alloSCT was 18.6 months (95% CI 9.6–NE) and 20.0 months (95% CI 10.3–24.1), respectively (Additional file 1: Fig. S4A and B).

At data cutoff, 12 (21%) of the 57 patients with CR/CRi were in ongoing remission without additional therapy; 14 (25%) proceeded to subsequent alloSCT (at data cutoff, seven were in remission, one relapsed, one initiated new anti-cancer therapy, and five had died); 8 (14%) proceeded to other anticancer therapies (Table S6; at data cutoff, three were in remission, two proceeded to subsequent alloSCT, three had died); 19 (33%) relapsed, three (5%) had died, and one was lost to follow-up. Median RFS among all treated patients with and without censoring at subsequent alloSCT was 11.7 months (95% CI 6.1–20.5) and 11.7 months (95% CI 6.1–20.5), respectively; and was 20.5 months (95% CI 11.7–NE) and 21.9 months (95% CI 12.3–26.0) for responders (*n* = 57), respectively (Additional file 1: Fig. S4C and D). Median OS was 25.4 months (95% CI 16.2–NE) for all treated patients and 47.0 months (95% CI 23.2–NE) for responders (Additional file 1: Fig. S4E).

### Impact of age and baseline bone marrow blasts percentages

Among phase 2 treated patients (*N* = 55) aged 18–25 (*n* = 12), 18–39 (*n* = 26), 40–59 (*n* = 20), and ≥ 60 (*n* = 9) years, CR/CRi rates were 67% (95% CI 35–90), 62% (95% CI 41–80), 70% (95% CI 46–88), and 100% (95% CI 66–100), respectively. Medians for DOR, RFS, and OS in each subgroup were largely consistent with the all-treated population (Table 1; Additional file 1: Fig. S5). Grade ≥ 3 CRS occurred in 25%, 23%, 20%, and 33% of patients aged 18–25, 18–39, 40–59, and ≥ 60 years, and grade ≥ 3 NEs occurred in 42%, 31%, 20%, and 22%, respectively.

The CR/CRi rates among patients with pre-KTE-X19 infusion (baseline) bone marrow blast percentages 0–5 (*n* = 5), > 5–25 (*n* = 10), > 25–50 (*n* = 11), > 50–75 (*n* = 10), and > 75–100 (*n* = 19) were 80% (95% CI 28–99), 90% (95% CI 55–100), 91% (95% CI 59–100), 80% (95% CI 44–97), and 42% (95% CI 20–67), respectively. Medians for DOR, RFS, and OS for most subgroups were largely consistent with the all-treated population; however, medians were lower for patients with > 75% baseline bone marrow blasts (Table 1; Additional file 1: Fig. S6). Grade ≥ 3 CRS occurred in 20%, 10%, 27%, 10%, and 37% of patients with baseline bone marrow blast percentages 0–5, > 5–25, > 25–50, > 50–75, and > 75–100; and grade ≥ 3 NEs occurred in 20%, 30%, 55%, 10%, and 16% of patients, respectively. Similar efficacy results observed in the pooled phase 1 and 2 (*N* = 78) analysis support the phase 2 subgroup findings by age and baseline bone marrow blast percentage (Table 1).

### Updated SCHOLAR-3 outcomes

As of July 23, 2021, forty-nine treated patients from phase 2 of ZUMA-3 were matched with 40 treated patients from historical clinical trials (SCA arms; Additional file 1: Supplemental Results and Fig. S7). Propensity-matching scores and baseline characteristics for the treated patients were previously reported [8].

**Fig. 3:**
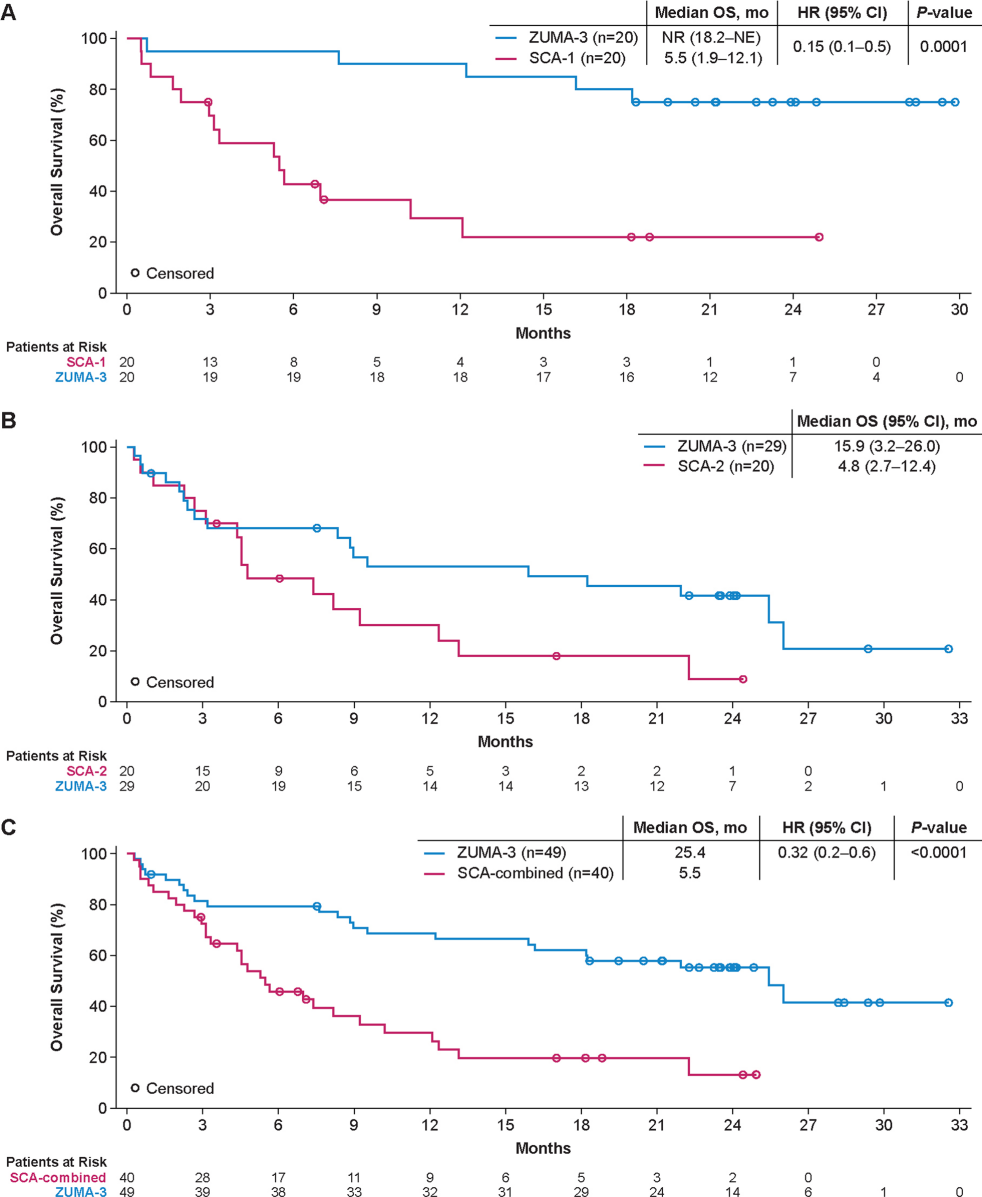
Overall survival of matched ZUMA-3 and SCHOLAR-3 treated patients. Kaplan**–**Meier OS estimates of ZUMA-3 and SCA patients who were previously naïve to blinatumomab and inotuzumab (SCA-1; **A**), patients who were previously treated with blinatumomab or inotuzumab (SCA-2, **B**) and all matched patients (SCA-combined; **C**). CR = complete remission. CRi = complete remission with incomplete hematological recovery. NE = not estimable

For matched patients previously naïve to blinatumomab and inotuzumab, the CR/CRi rates were 85% (95% CI 62.1–96.8) for treated ZUMA-3 patients (*n* = 20) and 35% (95% CI 15.4–59.2) for treated SCA-1 patients (*n* = 20; odds ratio, 10.5 [95% CI 2.3–48.7] *p* = 0.0031; Table 2). A comparison of CR/CRi rates by key patient subgroups is reported in Additional file 1: Fig. S8. Of the matched patients previously naïve to blinatumomab and inotuzumab, seven ZUMA-3 patients (35%) and four SCA-1 patients (20%) proceeded to subsequent alloSCT after treatment on clinical trial (Table 2). Median RFS was 20.5 months (95% CI 2.8–NE) for matched ZUMA-3 patients and 0.03 months (95% CI 0.0–4.6) for SCA-1 patients (hazard ratio [HR], 0.18 [95% CI 0.06–0.52]; *p* = 0.0004; Table 2 and Additional file 1: Fig. S9). Median OS was NR (95% CI 18.2–NE) for ZUMA-3 patients and 5.5 months (95% CI 1.9–12.1) for SCA-1 patients (HR 0.15 [95% CI 0.05–0.45]; *p* = 0.0001; Fig. 3A). The 18-month OS rate was 80% (95% CI, 55.1–92.0) for matched ZUMA-3 patients and 22% (95% CI, 6.0–44.3) for SCA-1 patients.

**Table 2:** Comparison of efficacy outcomes in matched patients who were previously naïve to blinatumomab and inotuzumab in ZUMA-3 and SCA-1

	Blinatumomab and inotuzumab-naïve patients	
	ZUMA-3 matched patients (*n* = 20)	SCA-1* (*n* = 20)
Overall CR/CRi rate, % (95% CI)	85.0 (62.1–96.8)	35.0 (15.4–59.2)
CR rate	75.0 (50.9–91.3)	30.0 (11.9–54.3)
Treatment difference (95% CI)	50.0 (17.9–73.7)	
Odds ratio (95% CI)	10.5 (2.3–48.7)	
*p* value	0.0031	
AlloSCT rate, % (95% CI)	35.0 (15.4–59.2)	20.0 (5.7–43.7)
Treatment difference (95% CI)	15.0 (-13.7–42.4)	
Odds ratio (95% CI)	2.2 (0.5–9.0)	
*p* value	0.4801	
Median RFS (95% CI), months	20.5 (2.8–NE)	0.0 (0.0–4.6)
Hazard ratio (95% CI)	0.18 (0.1–0.5)	
*p* value	0.0004	
Median OS (95% CI), months	NR (18.2–NE)	5.5 (1.9–12.1)
Hazard ratio (95% CI)	0.15 (0.1–0.5)	
*p* value	0.0001	

^*^SCA-1: SCHOLAR-3 patients who were previously naïve to blinatumomab and inotuzumab at enrollment in historical trials in which they may have received blinatumomab or inotuzumab

AlloSCT = allogeneic stem cell transplant. CI = confidence interval. CR = complete remission. CRi = complete remission with incomplete hematological recovery. NE = not estimable. NR = not reached. RFS = relapse-free survival. SCA = synthetic control arm

In patients previously treated with blinatumomab or inotuzumab, the median OS was 15.9 months (95% CI 3.2–26.0) for matched ZUMA-3 patients (*n* = 29) and 4.8 months (95% CI 2.7–12.4) for SCA-2 patients (*n* = 20; adjusted HR, 0.55 [95% CI 0.26–1.13]; Fig. 3B; Supplemental Results). Median OS for all matched patients was 25.4 months (95% CI 15.9–NE) for ZUMA-3 patients (*N* = 49) and 5.5 months (95% CI 3.3–9.2) for SCA-combined patients (*N* = 40; HR, 0.32 [95% CI 0.18–0.58]; *p* = 0.0001; Fig. 3C).

In the intention-to-treat analysis, 65 patients from ZUMA-3 Phase 2 were matched with 65 patients from historical control trials (SCA arms). For patients previously naïve to blinatumomab and inotuzumab, the CR/CRi rates were 72% (95% CI 50.6–87.9) for ZUMA-3 patients (*n* = 25) and 36% (95% CI 18.0–57.5) for SCA-1 patients (*n* = 25; odds ratio, 4.6 [95% CI 1.4–15.1] *p* = 0.0222; Table S7) and medians for OS were NR (95% CI, NE–NE) and 8.5 (95% CI, 4.2–20.3), respectively. In patients previously treated with blinatumomab or inotuzumab, the median OS was 9.7 months (95% CI 4.1–19.0) for matched ZUMA-3 patients (*n* = 40) and 4.7 months (95% CI 3.5–6.8) for SCA-2 patients (*n* = 40; HR, 0.66 [95% CI 0.37–1.17]; *p* = 0.1405. Median OS for all matched ITT patients was 23.1 months (95% CI 9.9–NE) for ZUMA-3 patients (*N* = 65) and 6.0 months (95% CI 4.2–7.3) for all SCA patients (*N* = 65; HR, 0.47 [95% CI 0.29–0.76]; *p* = 0.0011). Additional efficacy outcomes for the ITT population are reported in Table S7.

## Discussion

Despite the availability of new treatments, such as blinatumomab and inotuzumab, outcomes remain poor for adults with R/R B-ALL, with a median OS of less than 8 months following these therapies [2, 3]. With over 2 years of follow-up (median 26.8 months) in phase 2 of the ZUMA-3 study evaluating KTE-X19 in adult patients with R/R B-ALL, responses remained durable with a median DOR of 14.6 months for all responders and 20.0 months for those with CR, translating into a median OS of more than 2 years (25.4 months) in all patients and not reached in those with CR in a heavily pretreated population. Median DOR was extended without censorship of patients with subsequent alloSCT (18.6 months); however, small patient numbers limit the interpretability of these results. Studies to assess the impact of alloSCT after CAR T-cell therapy in this patient population are ongoing. Outcomes from an extended data set, including 23 patients in the phase 1 study who received the phase 2 dose of KTE-X19, supported the phase 2 findings.

After 2 years of follow-up in ZUMA-3, OS appeared extended compared with that reported for standard-of-care therapies, including blinatumomab, inotuzumab, or chemotherapy, in patients with R/R B-ALL [2, 3]. In addition, we conducted the retrospective historical external control study SCHOLAR-3 to contextualize ZUMA-3 findings in the R/R B-ALL treatment paradigm. Interestingly, prior exposure to blinatumomab or inotuzumab did not largely impact outcomes of patients treated in historical control arms in SCHOLAR-3, as median OS was less than 6 months, regardless of prior therapy status (blinatumomab/inotuzumab-treated or -naïve); though, patient numbers were small in these subgroups. In contrast, matched ZUMA-3 patients achieved a median OS of > 25 months, more than four times that of the historical control patients, highlighting a considerable benefit of KTE-X19 over standard-of-care therapies in this patient population; however, interpretation of these results should be cautioned due to the retrospective and external nature of the control group.

Response and OS benefits were observed across all age groups and percentage of baseline bone marrow blast subgroups. While patients ≥ 60 years of age appeared to receive the greatest benefit (100% CR/CRi rate and median OS NR), caution in interpretation is warranted due to small sample size (*n* = 9). In patients with > 75% percentage of pre-KTE-X19 infusion bone marrow blasts, overall CR/CRi rate (42.1%) and median OS (14.2 months) trended lower compared with the overall population. Despite differences in trial designs, eligibility criteria, and patient populations, these outcomes in ZUMA-3 compare favorably with the TOWER trial in which patients in the blinatumomab arm with ≥ 50% baseline bone marrow blasts achieved a 34.4% overall remission rate and a median OS of 5 months [2].

Long-term safety was favorable, with no new safety signals and no new onset of CAR T-cell therapy-specific AEs of interest, including CRS or neurological events. As of the new data cutoff, all cytopenias had resolved, with no late-onset infections or secondary malignancies, and one new grade 5 AE that was considered not treatment-related. At 24 months, CAR T cells were undetectable in all four evaluable patients in ongoing remission, suggesting that persistence of detectable CAR T cells in blood may not be required for durable responses, as previously reported in other disease types [10, 11].

A limitation of the ZUMA-3 study is the single-arm design; however, the SCHOLAR-3 analysis helped to contextualize the study results using blinded propensity-matched scoring to provide a robust analysis of ZUMA-3 efficacy outcomes in the context of historical standard-of-care therapies for R/R B-ALL. Although SCHOLAR-3 was retrospective in nature and conducted with a limited data set, the analyses were pre-specified and conducted by independent review. In addition, ZUMA-3 was conducted at multiple centers across Europe and North America with a large patient population, which was further extended in this analysis by a newly conducted independent central assessment of the additional 23 patients in phase 1 treated at the phase 2 dose.

## Conclusions

Together with the extended dataset including the additional phase 1 patients, these updated ZUMA-3 outcomes represent the longest follow-up of CAR T-cell therapy in a multicenter study of adult patients with R/R B-ALL to date and demonstrate that a single infusion of KTE-X19 resulted in durable outcomes with favorable long-term safety. Despite most patients being heavily pretreated, median OS was not yet reached in patients who achieved CR after > 2 years of median follow-up. Furthermore, KTE-X19 appears to improve outcomes compared to historical standard-of-care therapies and helps to address an unmet need for patients with R/R B-ALL.

## Supplementary Information

**Additional file 1**. Supplementary Appendix.

## Data Availability

Kite is committed to sharing clinical trial data with external medical experts and scientific researchers in the interest of advancing public health, and access can be requested by contacting medinfo@kitepharma.com.

## Abbreviations

AE: Adverse event
alloSCT: Allogeneic stem cell transplant
AUC: Area under the curve
B-ALL: B-precursor acute lymphoblastic leukemia
BFBM: Blast-free hypoplastic or aplastic bone marrow
CAR: Chimeric antigen receptor
CR: Complete remission
Cri: Complete remission with incomplete hematological recovery
CRS: Cytokine release syndrome
DOR: Duration of remission
GVHD: Graft vs host disease
ITT: Intention-to-treat
KTE-X19: Brexucabtagene autoleucel
MRD: Minimal residual disease
NE: Not estimable
NR: Not reached
OS: Overall survival
RFS: Relapse-free survival
R/R: Relapsed or refractory
SCA: Synthetic control arm
