# Supplementary material for: Two-year follow-up of KTE-X19 in patients with relapsed or refractory adult B-cell acute lymphoblastic leukemia in ZUMA-3 and its contextualization with SCHOLAR-3, an external historical control study
Source: J Hematol Oncol. 2022 Dec 10;15:170. doi: 10.1186/s13045-022-01379-0 (PMC9734710; doi:10.1186/s13045-022-01379-0)
Supplement: Supplementary file 1 — Additional file 1. Supplementary Appendix. [file 13045_2022_1379_MOESM1_ESM.pdf]

SUPPLEMENTAL APPENDIX

Table of Contents

SUPPLEMENTAL APPENDIX 1

Supplementary Methods 3

Eligibility criteria 3

Procedures 3

Outcomes 3

Statistical analyses 3

SCHOLAR-3 3

Supplementary Results 3

Patients 3

Efficacy outcomes with long-term follow-up 4

SCHOLAR-3 participants 4

Table S1: Baseline characteristics by age and baseline bone marrow blast percentage in phase 2 treated patients (N=55) 5

Table S2: Baseline characteristics for all phase 1 and 2 patients treated with pivotal dose (N=78) and by age and baseline bone marrow blast percentage 7

Table S3: Summary of subsequent anti-cancer therapies for patients who received other anti-cancer therapies while in remission following KTE-X19 (N=55) 9

Table S4: Updated efficacy outcomes based on central assessment in all enrolled phase 2 patients (N=71) 10

Table S5: Summary of peak and AUC CAR T cells in blood by age 11

Table S6: Summary of subsequent anti-cancer therapies for patients who received other anti-cancer therapies while in remission following KTE-X19 (N=78) 12

Table S7: Comparison of ITT efficacy outcomes in matched patients who were previously naïve to blinatumomab and inotuzumab or previously treated with blinatumomab or inotuzumab enrolled in ZUMA-3 or historical trials (SCA-1 and SCA-2) 13

Supplementary Figures 15

Figure S1. Kaplan-Meier curve of RFS without censoring at alloSCT in phase 2 treated patients (N=55). 15

Figure S2. Median (Q1, Q3) CAR T-cell levels over time in phase 2 treated patients. 16

Figure S3. Peak (A) and AUC (B) CAR T-cell levels in blood by ongoing response in evaluable phase 2 patients (n=35). 17

Figure S4. DOR with (A) and without censoring (B), RFS with (C) and without censoring (D) and OS (E) KM curves for combined analysis (N=78). 18

Figure S5. Kaplan-Meier estimates of OS by age subgroups (18–25, 18–39, 40–59, ≥60 years) in phase 2 treated patients (N=55). 23

Figure S6. Kaplan-Meier estimates of OS by pre-KTE-X19 infusion bone marrow blast percentage subgroups (≤5%, >5%–25%, >25%–50%, >50%–75%, and >75%–100%) in phase 2 treated patients (N=55). 23

Figure S7. Treatment summary in SCHOLAR-3 cohorts 24

Figure S8. Comparison of CR/CRi rates between matched blinatumomab and inotuzumab-naïve ZUMA-3 patients (n=20) and SCA-1 patients (n=20) by key subgroups. 26

Figure S9. Kaplan-Meier curves of RFS for matched blinatumomab and inotuzumab-naïve ZUMA-3 patients (n=20) and SCA-1 patients (n=20). 27

Supplementary References 28

Supplementary Methods

Eligibility criteria

Complete eligibility requirements were previously reported.[1,2] Relapsed or refractory disease was defined as primary refractory, first relapse after remission of 12 months or less, relapsed or refractory after two or more previous lines of systemic therapy, or relapsed or refractory after allogeneic stem cell transplant (alloSCT).

Procedures

Patients were permitted to receive one additional KTE-X19 infusion, provided they had achieved remission (complete remission [CR], CR with partial hematologic recovery [CRh], or complete remission with incomplete hematological recovery [CRi]) after the initial KTE-X19 infusion at ≥3-month disease assessment and subsequently progressed (>5% bone marrow blasts or progression of extramedullary disease per local assessment) with documented CD19 tumor expression in bone marrow or peripheral blood. If CD19 expression was quantified, then there must be ≥90% CD19 positive blasts to proceed to retreatment.

Disease and adverse event monitoring and toxicity management were previously described.[1]

Outcomes

Definitions for duration of remission (DOR), overall survival (OS), and relapse-free survival (RFS) were previously reported.[1] Briefly, DOR was defined as the time from reaching CR or CRi (central assessment) to relapse or death without documented relapse. The calculation of DOR did not include disease assessments occurring after new anticancer therapies, including alloSCT. RFS was defined as the time from infusion of KTE-X19 to the date of relapse or death from any cause. Patients were evaluated as having an RFS event at day 0 if they did not achieve CR or CRi as of the data cutoff date; as such, patients in the ITT population who were not infused would also have been considered to have an event at day 0. For duration of remission and relapse-free survival, sensitivity analyses were performed in which disease assessments obtained after alloSCT were included in the derivation of duration of remission and relapse-free survival.

Statistical analyses

Analyses sets:

- Phase 2 intention-to-treat (ITT) analysis set (N=71): Consisted of all patients enrolled in Phase 2
- Phase 2 treated patients (modified ITT analysis set [mITT]; N=55): Consisted of all patients enrolled in Phase 2 and treated with KTE-X19
- Phase 1 and phase 2 combined (N=78): Consisted of all patients treated in phase 1 (n=23) and phase 2 (n=55) at the recommended phase 2 dose of KTE-X19 ($1X10^6$ CAR T cells/kg)

SCHOLAR-3

The SCA-1 data pool included the following patients from historical clinical trials: 1) patients who were blinatumomab or inotuzumab treatment-naïve at trial enrolment and had received standard of care or inotuzumab as initial study treatment; and 2) 20% of patients who were blinatumomab or inotuzumab treatment-naïve at trial enrolment and received blinatumomab as initial study treatment (randomly selected). The SCA-2 data pool included the following patients from historical clinical trials: 1) patients who previously received blinatumomab or inotuzumab at trial enrolment and received standard of care or inotuzumab as initial study treatment; and 2) remaining 80% of patients who were blinatumomab or inotuzumab treatment-naïve at trial enrolment and received blinatumomab as initial study treatment, and then subsequently switched study treatment from blinatumomab to other standard of care.

Supplementary Results

Patients

Most baseline patient and disease characteristics for phase 2 treated patients (N=55) were similar between subgroups, although some differences were observed (**Table S1**). The proportion of males was lower in the group aged 40–59 years compared to other age subgroups (45% vs 65%–92%) and patients ≥60 years of age more frequently had Philadelphia chromosome-positive disease compared to other age subgroups (67% vs 0%–30%). In

addition, patients ≤39 years of age were more likely to be primary refractory than older patients (50%–58% vs 11%–20%) and more likely to have a higher percentage of bone marrow blasts at baseline than older patients (medians 70.5–71.5 vs 46–50)**;** however, patient numbers in each group were limited.

The group with >5% to 25% baseline bone marrow blasts had a greater proportion of patients who were ≥60 years of age (60% vs 5%–10%) as well as a greater proportion of patients who had Philadelphia chromosome-positive disease (50% vs 10%–40%) compared with other subgroups. Among patients with ≤5% and >50% to 75% of baseline bone marrow blasts, there were fewer males compared with other subgroups (40% vs 58%–80%). In addition, there was a wide range of median number of prior therapies between subgroups, with patients having ≤5% baseline bone marrow blasts having the highest median (4) and patients with >50% to 75% baseline bone marrow blasts having the lowest median (1.5). Similar trends were observed in the extended phase 1 and 2 analyses (N=78).

Efficacy outcomes with long-term follow-up

For phase 2 treated patients, the RFS rates (95% CI) at 18 months with and without censoring patients at subsequent alloSCT were 35.4% (20.5–50.6) and 41.8% (28.0–55.0), respectively (**Figure S2**), and the overall survival rate at 24 months was 55.7% (41.2–68.1; F**igure S1**).

SCHOLAR-3 participants

The SCHOLAR-3 long-term follow-up analyses (data cutoff date: July 23, 2021) included updated outcomes for 89 matched treated patients (ZUMA-3, N=49; SCA, N=40). Originally, 29 patients were included in the SCA-2 control arm; however, 9 patients were found to have important protocol deviations; namely, that they did not have documented relapse prior to starting a subsequent therapy. As such, these 9 patients were excluded, and the SCA-2 arm subsequently consisted of 20 patients for this analysis. In addition, a new analysis of 130 matched ITT patients (ZUMA-3, N=65; SCA, N=65) was conducted. Blinatumomab and inotuzumab-naïve arms included 25 ZUMA-3 patients (four patients were not infused with KTE-X19) matched to 25 SCA-1 patients. Blinatumomab- and inotuzumab-treated arms included 40 ZUMA-3 patients (ten patients were not infused with KTE-X19) matched to 40 SCA-2 patients.

Table S1: Baseline characteristics by age and baseline bone marrow blast percentage in phase 2 treated patients (N=55)

	Age, years				Baseline BM Blasts				
**Characteristic**	**18–25 (n=12)**	**18–39 (n=26)**	**40–59 (n=20)**	**≥60 (n=9)**	**≤5% (n=5)**	**>5%–25% (n=10)**	**>25%–50% (n=11)**	**>50%–75% (n=10)**	**>75%–100% (n=19)**
Age, median (range), years	22.0 (19–25)	27.5 (19–39)	48.5 (40–59)	66.0 (62–84)	48.0 (36–53)	63.5 (21–84)	31.0 (21–66)	41.5 (21–71)	36.0 (19–68)
≥60 years, n (%)	0	0	0	9 (100)	0	6 (60)	1 (9)	1 (10)	1 (5)
Male, n (%)	11 (92)	17 (65)	9 (45)	7 (78)	2 (40)	8 (80)	8 (73)	4 (40)	11 (58)
ECOG PS of 1, n (%)	8 (67)	18 (69)	16 (80)	5 (56)	5 (100)	6 (60)	5 (45)	8 (80)	15 (79)
Philadelphia chromosome-positive, n (%)	0	3 (12)	6 (30)	6 (67)	2 (40)	5 (50)	2 (18)	1 (10)	5 (26)
Baseline extramedullary disease, n (%)	2 (17)	5 (19)	1 (5)	0	0	0	4 (36)	1 (10)	1 (5)
CNS-1 disease at baseline, n (%)	12 (100)	26 (100)	20 (100)	9 (100)	5 (100)	10 (100)	11 (100)	10 (100)	19 (100)
Number of prior therapies, median (range)	2.5 (1–4)	3.0 (1–8)	2.0 (1–5)	2.0 (1–4)	4.0 (1–4)	2.0 (1–4)	2.0 (1–3)	1.5 (1–5)	3.0 (2–8)
≥3 prior lines of therapy, n (%)	6 (50)	14 (54)	8 (40)	4 (44)	3 (60)	3 (30)	4 (36)	4 (40)	12 (63)
Prior blinatumomab, n (%)	6 (50)	13 (50)	9 (45)	3 (33)	2 (40)	5 (50)	3 (27)	4 (40)	11 (58)
Prior inotuzumab ozogamicin, n (%)	2 (17)	4 (15)	6 (30)	2 (22)	1 (20)	1 (10)	1 (9)	3 (30)	6 (32)
Prior allogeneic SCT, n (%)	4 (33)	12 (46)	9 (45)	2 (22)	3 (60)	3 (30)	3 (27)	3 (30)	11 (58)
Relapsed/refractory subgroup, n (%)									
Primary refractory	7 (58)	13 (50)	4 (20)	1 (11)	0	3 (30)	4 (36)	3 (30)	8 (42)
Relapsed or refractory to second or greater lines of therapy	8 (67)	18 (69)	17 (85)	8 (89)	4 (80)	8 (80)	9 (82)	4 (40)	18 (95)
First relapse with remission ≤12 months	2 (17)	5 (19)	8 (40)	3 (33)	2 (40)	4 (40)	3 (27)	5 (50)	2 (11)

Relapsed or refractory post-alloSCT	4 (33)	12 (46)	10 (50)	2 (22)	4 (80)	3 (30)	3 (27)	3 (30)	11 (58)
BM blasts at screening									
Median (range), %	77 (7.5–97)	74 (7.5–100)	46 (5–96)	50 (6–96)	89.0 (24–100)	15.0 (5–88)	24.0 (6–92)	50.0 (12–70)	80.0 (25–97)
≤5%, n (%)	0	0	0	0	0	0	0	0	0
>5%– 25%, n (%)	3 (25)	6 (23)	8 (40)	2 (22)	1 (20)	6 (60)	6 (55)	2 (20)	1 (5)
M3 BM involvement (>25% blasts), n (%)	9 (75)	20 (77)	12 (60)	7 (77)	4 (80)	4 (40)	5 (45)	8 (80)	18 (95)
BM blasts at baseline									
Median (range), %	70.5 (7.2–97)	71.5 (5–97)	54.0 (0–98)	17.0 (6–96)	2.0 (0–5)	10.0 (6–25)	45.0 (28–50)	65.0 (58–73)	90.0 (7798)
≤5%, n (%)	0	1 (4)	4 (20)	0	5 (100)	0	0	0	0
>5%– 25%, n (%)	1 (8)	2 (8)	2 (10)	6 (67)	0	10 (100)	0	0	0
M3 BM involvement (>25% blasts), n (%)	11 (92)	23 (88)	14 (70)	3 (33)	0	0	11 (100)	10 (100)	19 (100)
BM blasts at preconditioning after bridging chemotherapy									
Median (range), %	50.0 (7.2–93)	67.5 (5–93)	54.0 (0–98)	20.5 (6-65)	2.0 (0–5)	10.0 (6–25)	45.0 (28–50)	65.0 (58–73)	90.0 (77–98)
≤5%, n (%)	0	1 (4)	4 (20)	0	5 (100)	0	0	0	0
>5%– 25%, n (%)	1 (8)	1 (4)	2 (10)	4 (44)	0	7 (70)	0	0	0
M3 BM involvement (>25% blasts), n (%)	8 (67)	20 (77)	12 (60)	2 (22)	0	0	10 (91)	9 (90)	15 (79)
Missing	3 (25)	4 (15)	2 (10)	3 (33)	0	3 (30)	1 (9)	1 (10)	4 (21)

BM=bone marrow. CNS=central nervous system. ECOG=Eastern Cooperative Oncology Group. PS=performance status. SCT=stem cell transplant. Baseline is defined as the last assessment prior to the start of conditioning chemotherapy.

Table S2: Baseline characteristics for all phase 1 and 2 patients treated with pivotal dose (N=78) and by age and baseline bone marrow blast percentage

Characteristic	All Phase 1 and 2 Patients (N=78)	Age, years				Baseline BM Blasts				
		18-25 (n=15)	18-39 (n=36)	40-59 (n=27)	≥60 (n=15)	≤5% (n=8)	>5%– 25% (n=14)	>25%–50% (n=12)	>50%–75% (n=14)	>75%–100% (n=30)
Age, median (range), years	42.5 (18–84)	22.0 (18–25)	27.5 (18–39)	49.0 (40–59)	68.0 (60–84)	50.5 (36–68)	63.0 (21–84)	35.5 (21–66)	41.5 (21–71)	36.5 (18–77)
≥60 years, n (%)	15 (19)	0	0	0	15 (100)	2 (25)	8 (57)	1 (8)	2 (14)	2 (7)
Male, n (%)	42 (54)	12 (80)	21 (58)	13 (48)	8 (53)	3 (38)	9 (64)	8 (67)	6 (43)	16 (53)
ECOG PS of 1, n (%)	56 (72)	11 (73)	26 (72)	21 (78)	9 (60)	7 (88)	10 (71)	6 (50)	10 (71)	23 (77)
Philadelphia chromosome-positive, n (%)	17 (22)	1 (7)	5 (14)	6 (22)	6 (40)	2 (25)	6 (43)	2 (17)	2 (14)	5 (17)
Baseline extramedullary disease, n (%)	9 (12)	2 (13)	6 (17)	1 (4)	2 (13)	0	2 (14)	4 (33)	2 (14)	1 (3)
CNS-1 disease at baseline, n (%)	78 (100)	15 (100)	36 (100)	27 (100)	15 (100)	8 (100)	14 (100)	12 (100)	14 (100)	30 (100)
Number of prior therapies, median (range)	2 (1–8)	3.0 (1–4)	3.0 (1–8)	2.0 (1–7)	2.0 (1–5)	2.5 (1–4)	2.0 (1–5)	2.0 (1–3)	2.0 (1–5)	3.0 (1–8)
≥3 prior lines of therapy, n (%)	37 (47)	8 (53)	19 (53)	11 (41)	7 (47)	4 (50)	5 (36)	5 (42)	6 (43)	17 (57)
Prior blinatumomab	38 (49)	8 (53)	19 (53)	12 (44)	7 (47)	3 (38)	7 (50)	4 (33)	7 (50)	17 (57)
Prior inotuzumab ozogamicin	17 (22)	3 (20)	5 (14)	8 (30)	4 (27)	1 (13)	2 (14)	2 (17)	3 (21)	9 (30)
Prior allogeneic SCT	29 (37)	5 (33)	15 (42)	11 (41)	3 (20)	4 (50)	5 (36)	3 (25)	5 (36)	12 (40)
Relapsed/refractory subgroup, n (%)										
Primary refractory	24 (31)	7 (47)	15 (42)	7 (26)	2 (13)	0	5 (36)	4 (33)	3 (21)	12 (40)
Relapsed or refractory to second or greater lines of therapy	60 (77)	11 (73)	26 (72)	21 (78)	13 (87)	6 (75)	10 (71)	10 (83)	8 (57)	26 (87)
First relapse with remission ≤12 months	22 (28)	2 (13)	6 (17)	10 (37)	6 (40)	5 (63)	5 (36)	3 (25)	5 (36)	4 (13)

Relapsed or refractory post-allo-SCT	30 (38)	5 (33)	15 (42)	12 (44)	3 (20)	5 (63)	5 (36)	3 (25)	5 (36)	12 (40)
BM blasts at screening										
Median (range), %	62 (5–100)	76.0 (7.5–97)	71.0 (7.5–100)	61.0 (5–96)	50.0 (5–96)	76.5 (5–100)	15.0 (5–88.1)	32.0 (6–92)	55.5 (12–97)	80.0 (25–97)
≤5%, n (%)	1 (1)	0	0	0	1 (7)	1 (13)	0	0	0	0
>5%–25%, n (%)	19 (24)	3 (20)	7 (19)	8 (30)	4 (27)	1 (13)	9 (64)	6 (50)	2 (14)	1 (3)
M3 BM involvement (>25% blasts), n (%)	58 (74)	12 (80)	29 (81)	19 (70)	10 (67)	6 (75)	5 (36)	6 (50)	12 (86)	29 (97)
BM blasts at baseline										
Median (range), %	63 (0–98)	76.0 (7.2–97)	74.5 (5–97)	58.0 (0–98)	16.0 (2–96)	2.0 (0–5)	10.4 (6–25)	43.1 (28–50)	65.0 (51–73)	90.0 (76–98)
≤5%, n (%)	8 (10)	0	1 (3)	5 (19)	2 (13)	8 (100)	0	0	0	0
>5%–25%, n (%)	14 (18)	1 (7)	4 (11)	2 (7)	8 (53)	0	14 (100)	0	0	0
M3 BM involvement (>25% blasts), n (%)	56 (72)	14 (93)	31 (86)	20 (74)	5 (33)	0	0	12 (100)	14 (100)	30 (100)
BM blasts at preconditioning after bridging chemotherapy										
Median (range), %	65 (0–98)	65.0 (7.2–93)	75.0 (5–97)	58.0 (0–98)	13.4 (2–80)	2.0 (0–5)	10.0 (6–25)	41.2 (28–50)	65.0 (51–73)	90.0 (77–98)
≤5%, n (%)	7 (9)	0	1 (3)	4 (15)	2 (13)	7 (88)	0	0	0	0
>5% to 25%, n (%)	9 (12)	1 (7)	2 (6)	2 (7)	5 (33)	0	9 (64)	0	0	0
M3 BM involvement (>25% blasts), n (%)	47 (60)	10 (67)	27 (75)	17 (63)	3 (30)	0	0	11 (92)	11 (79)	25 (83)
Missing	15 (19)	4 (27)	6 (17)	4 (15)	5 (33)	1 (13)	5 (36)	1 (8)	3 (21)	5 (17)

BM=bone marrow. CNS=central nervous system. ECOG=Eastern Cooperative Oncology Group. PS=performance status. SCT=stem cell transplant. Baseline is defined as the last assessment prior to the start of conditioning chemotherapy.

Table S3: Summary of subsequent anti-cancer therapies for patients who received other anti-cancer therapies while in remission following KTE-X19 (N=55)

	**Phase 2 (N=55)**
Patients who proceeded to new anti-cancer therapy while in remission	6 (11)
Dexamethasone	3 (5)
Inotuzumab	3 (5)
Cyclophosphamide	2 (4)
Inotuzumab Ozogamicin	2 (4)
Vincristine	2 (4)
Antithymocyte immunoglobulin (rabbit)	1 (2)
Blinatumomab	1 (2)
Cytarabine	1 (2)
Fludarabine Phosphate	1 (2)
Haploid Allo Tx*	1 (2)
Hydrocortisone	1 (2)
Inotuzumab Ozogamicin	1 (2)
Investigational Antineoplastic Drugs	1 (2)
Melphalan	1 (2)
Methotrexate	1 (2)
Ponatinib	1 (2)
Ponatinib Hydrochloride	1 (2)
Rituximab	1 (2)
Thiotepa	1 (2)

* Indicates reported name of therapy.

Table S4: Updated efficacy outcomes based on central assessment in all enrolled phase 2 patients (N=71)

**n (%)**	**N=71**
Overall CR/CRi	39 (55)
CR	31 (44)
CRi	8 (11)
Blast-free hypoplastic or aplastic bone marrow	4 (6)
No response	11 (15)
Unknown or not evaluable	17 (24)
Median DOR (95% CI), months	14.6 (9.4–NE)
Median RFS (95% CI), months	3.7 (0.0–12.9)
Median OS (95% CI), months	23.1 (10.4–NE)

CR=complete remission. CRi=complete remission with incomplete hematological recovery. DOR=duration of remission. NE=not estimable. OS=overall survival. RFS=relapse-free survival.

Table S5: Summary of peak and AUC CAR T cells in blood by age

	**18–25 years (n=12)**	**18–39 years (n=26)**	**40–59 years (n=20)**	**≥60 years (n=9)**
**$AUC_{0\text{-}28}$ (cells/µL*days)**				
n	11	23	19	8
Median (range)	126.2 (1.4–2,624.5)	90.5 (0.0–19,390.4)	199.8 (0.0–2212.0)	436.1 (222.7– 698.1)
**Peak (cells/µL)**				
n	11	23	19	8
Median (range)	9.0 (0.4–243.2)	6.0 (0.0–1533.4)	18.8 (0.0–190.7)	37.6 (16.0–86.8)
**Time To Peak (days)**				
n	11	23	19	8
Median (range)	15 (8–30)	15 (7–30)	15 (8–32)	14 (8–15)

AUC=area under the curve. CAR=chimeric antigen receptor.
$AUC_{0\text{-}28}$ is defined as the AUC in a graph of number of CAR T cells in blood against scheduled visit from Day 0 to Day 28. Peak is defined as the maximum number of CAR T cells in blood measured after infusion. Time-to-peak is defined as the number of days from KTE-X19 infusion to the date when the number of CAR T cells in blood first reached the maximum post-baseline level.

Table S6: Summary of subsequent anti-cancer therapies for patients who received other anti-cancer therapies while in remission following KTE-X19 (N=78)

	**Phase 1 and 2 (N=78)**
Patients who proceeded to new anti-cancer therapy while in remission	8 (10)
Cyclophosphamide	4 (5)
Dexamethasone	4 (5)
Inotuzumab	3 (4)
Vincristine	3 (4)
Inotuzumab Ozogamicin	2 (3)
Antithymocyte immunoglobulin (rabbit)	1 (1)
Blinatumomab	1 (1)
Blinatumomab*	1 (1)
Cytarabine	1 (1)
Etoposide	1 (1)
Fludarabine	1 (1)
Fludarabine Phosphate	1 (1)
Haploid Allo Tx*	1 (1)
Hydrocortisone	1 (1)
Inotuzumab Ozogamicin	1 (1)
Investigational Antineoplastic Drugs	1 (1)
Melphalan	1 (1)
Methotrexate	1 (1)
Ponatinib	1 (1)
Ponatinib Hydrochloride	1 (1)
Rituximab	1 (1)
Thiotepa	1 (1)
Tisagenlecleucel-T	1 (1)

* Indicates reported name of therapy.

Table S7: Comparison of ITT efficacy outcomes in matched patients who were previously naïve to blinatumomab and inotuzumab or previously treated with blinatumomab or inotuzumab enrolled in ZUMA-3 or historical trials (SCA-1 and SCA-2)

	**Blinatumomab and inotuzumab-naïve patients**	
	**ZUMA-3 (N=25)**	**SCA-1* (N=25)**
Overall CR/CRi rate at 24 weeks, % (95% CI)	72 (50.6–87.9)	36 (18.0–57.5)
CR rate	64 (42.5–82.0)	32 (14.9–53.5)
Treatment difference (95% CI)	36 (7.4–60.4)	
Odds ratio (95% CI)	4.6 (1.4–15.1)	
p value	0.0222	
alloSCT rate, % (95% CI)	24 (9.4–45.1)	32 (14.9–53.5)
Treatment difference (95% CI)	–8 (–33.0–17.7)	
Odds ratio (95% CI)	0.7 (0.2–2.3)	
p value	0.7536	
Median OS (95% CI), months	NR (NE–NE)	8.5 (4.2–20.3)
Hazard ratio (95% CI)	0.21 (0.08–0.55)	
p value	0.0006	
Median RFS (95% CI), months	11.5 (3.0–NE)	0.0 (0.0–4.6)
Hazard ratio (95% CI)	0.45 (0.21–0.95)	
p value	0.0337	
	**Blinatumomab or inotuzumab treated patients**	
	**ZUMA–3 (N=40)**	**SCA-2 (N=40)**
Median OS (95% CI), months	9.7 (4.1–19.0)	4.7 (3.5–6.8)
Hazard ratio (95% CI)	0.66 (0.37–1.17)	

p value	0.1405	
	**All matched patients**	
	**ZUMA-3 target group–1 and group–2 combined (N=65)**	**SCA-3 (SCA-1 and SCA-2 combined) (N=65)**
Median OS (95% CI), months	23.1 (9.9–NE)	6.0 (4.2–7.3)
Hazard ratio (95% CI)	0.47 (0.29–0.76)	
p value	0.0011	

*SCA-1: SCHOLAR-3 patients who were previously naïve to blinatumomab and inotuzumab at enrollment in historical trials in which they may have received blinatumomab or inotuzumab. Remission responses from target group are based on independent review and remission responses from SCA are based on investigator review. The 95% CIs for rate and rate difference are exact confidence limits, and p value is estimated for rate difference from Fisher's exact test. The 95% confidence interval for odds ratio is based on Wald statistics from logistic regression. CR=complete remission. CRi=CR with incomplete hematologic recovery. ITT=intention-to-treat. NE=not estimable. NR= not reached. OS=overall survival. SCA=synthetic control arm.

Supplementary Figures

Figure S1. Kaplan-Meier curve of RFS without censoring at alloSCT in phase 2 treated patients (N=55).

alloSCT=allogeneic stem cell transplant. CR=complete remission. CRi=CR with incomplete hematological recovery. NE=not estimable. RFS=relapse-free survival. SCT=stem cell transplant.

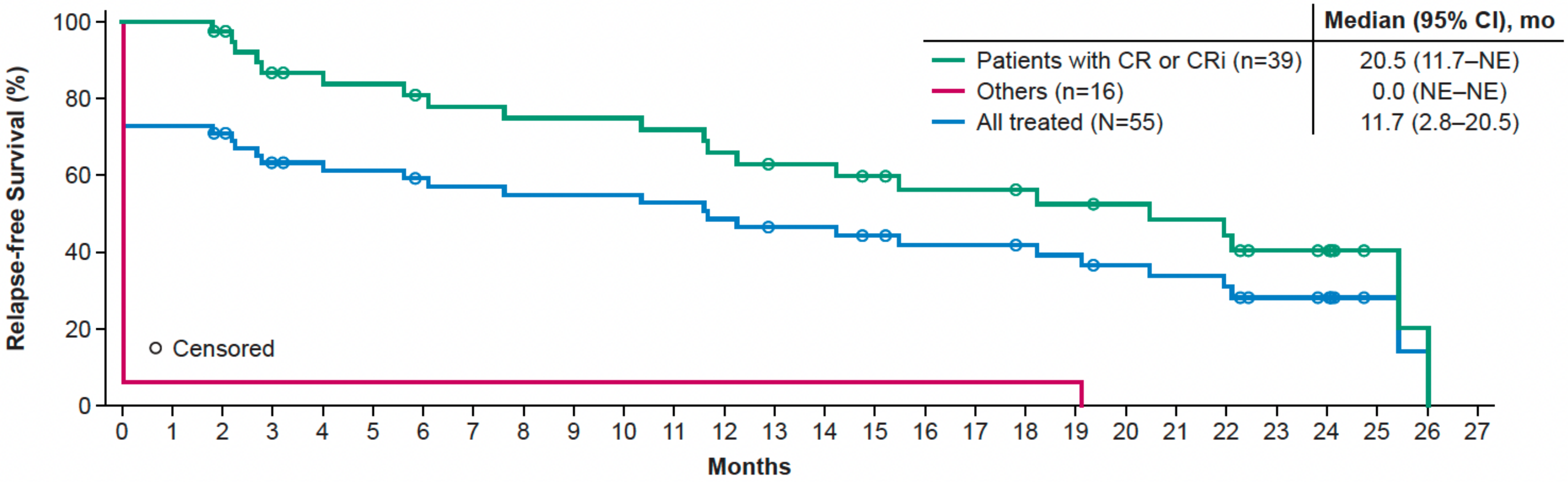

	0	1	2	3	4	5	6	7	8	9	10	11	12	13	14	15	16	17	18	19	20	21	22	23	24	25	26	27
CR+CRi at risk	39	39	37	31	30	29	27	26	25	25	25	24	22	20	20	18	16	16	15	14	13	12	11	8	7	2	1	0
(CR+CRi censored)	(0)	(0)	(1)	(3)	(4)	(4)	(5)	(5)	(5)	(5)	(5)	(5)	(5)	(6)	(6)	(7)	(8)	(8)	(9)	(9)	(10)	(10)	(10)	(12)	(13)	(18)	(18)	(18)
Others at risk	16	1	1	1	1	1	1	1	1	1	1	1	1	1	1	1	1	1	1	1	0	0	0	0	0	0	0	0
(Others censored)	(0)	(0)	(0)	(0)	(0)	(0)	(0)	(0)	(0)	(0)	(0)	(0)	(0)	(0)	(0)	(0)	(0)	(0)	(0)	(0)	(0)	(0)	(0)	(0)	(0)	(0)	(0)	(0)
All Dosed at risk	55	40	38	32	31	30	28	27	26	26	26	25	23	21	21	19	17	17	16	15	13	12	11	8	7	2	1	0
(All Dosed censored)	(0)	(0)	(1)	(3)	(4)	(4)	(5)	(5)	(5)	(5)	(5)	(5)	(5)	(6)	(6)	(7)	(8)	(8)	(9)	(9)	(10)	(10)	(10)	(12)	(13)	(18)	(18)	(18)

Figure S2. Median (Q1, Q3) CAR T-cell levels over time in phase 2 treated patients.

[a] Detected by polymerase chain reaction. CAR=chimeric antigen receptor.

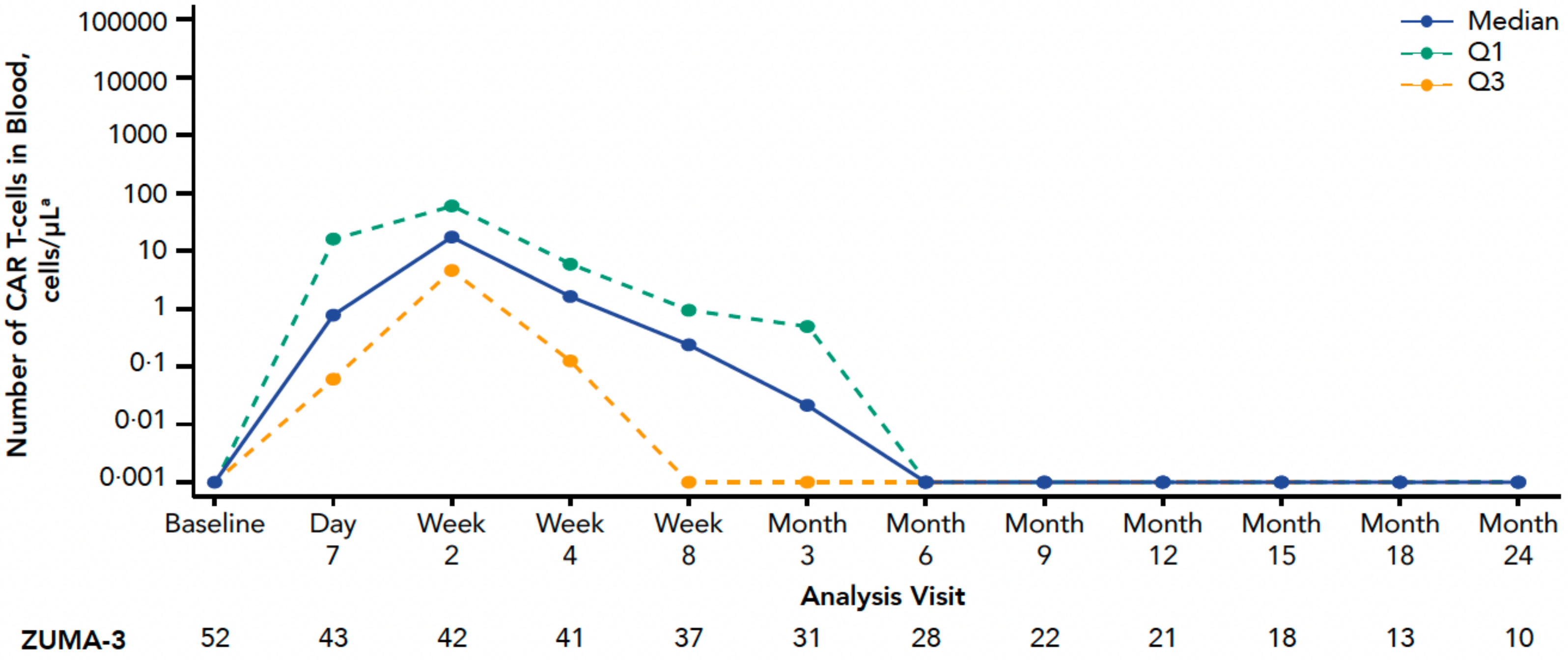

Figure S3. Peak (A) and AUC (B) CAR T-cell levels in blood by ongoing response in evaluable phase 2 patients (n=35).

[a] Patients had achieved a CR/CRi and then relapsed. AUC=area under the curve. CAR=chimeric antigen receptor. CR=complete response. CRi=CR with incomplete hematological recovery.

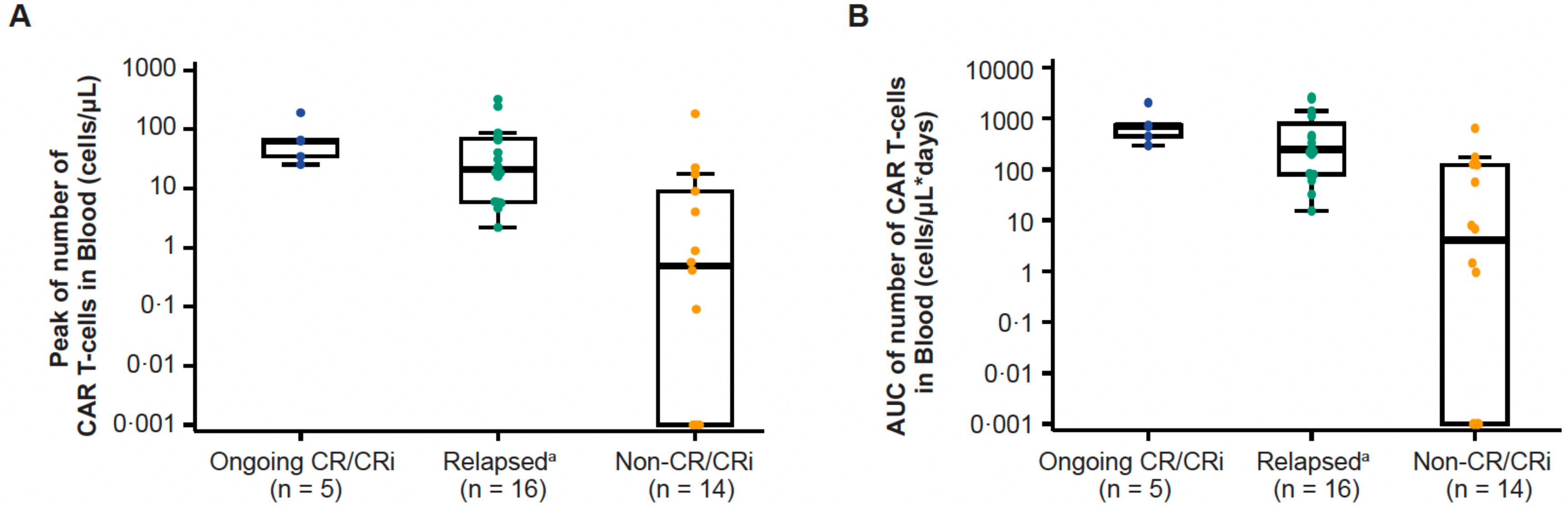

Figure S4. DOR with (A) and without censoring (B), RFS with (C) and without censoring (D) and OS (E) KM curves for combined analysis (N=78).

CR=complete remission. CRi=CR with incomplete hematological recovery. DOR=duration of response. KM=Kaplan-Meier. NE=not estimable. RFS=relapse-free survival.

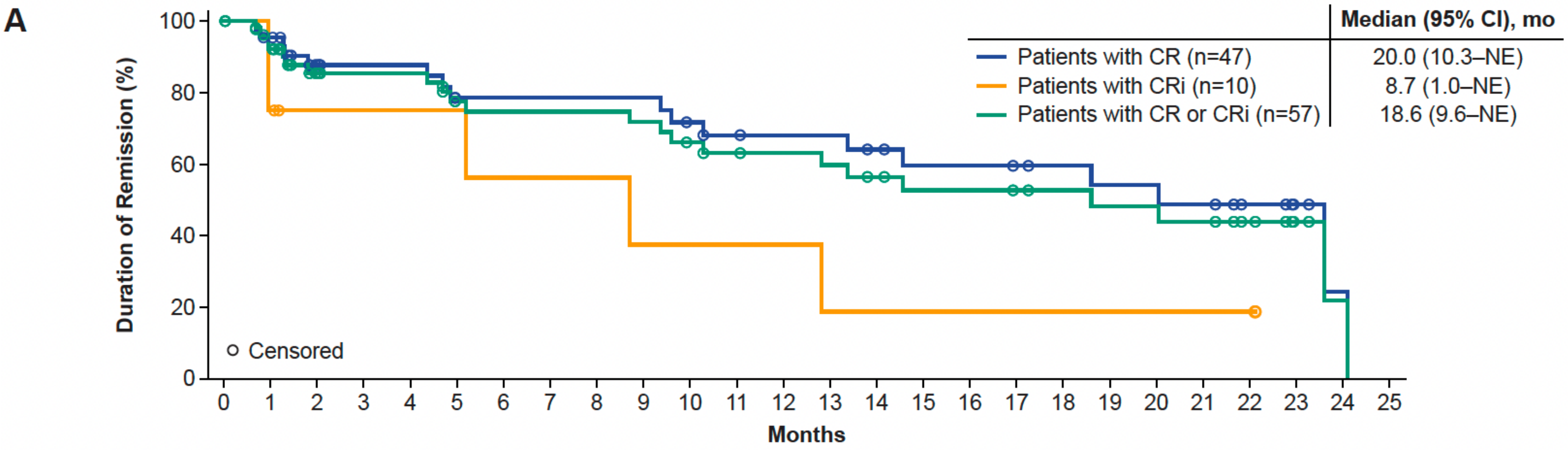

CR at risk	47	40	31	29	29	23	23	23	23	23	20	18	17	17	15	13	13	12	11	10	10	9	6	3	1	0
(CR censored)	(0)	(5)	(11)	(13)	(13)	(16)	(16)	(16)	(16)	(16)	(17)	(18)	(19)	(19)	(20)	(21)	(21)	(22)	(23)	(23)	(23)	(23)	(26)	(29)	(30)	(30)
CRi at risk	10	6	4	4	4	4	3	3	3	2	2	2	2	1	1	1	1	1	1	1	1	1	1	0	0	0
(CRi censored)	(0)	(2)	(4)	(4)	(4)	(4)	(4)	(4)	(4)	(4)	(4)	(4)	(4)	(4)	(4)	(4)	(4)	(4)	(4)	(4)	(4)	(4)	(4)	(5)	(5)	(5)
CR+CRi at risk	57	46	35	33	33	27	26	26	26	25	22	20	19	18	16	14	14	13	12	11	11	10	7	3	1	0
(CR+CRi censored)	(0)	(7)	(15)	(17)	(17)	(20)	(20)	(20)	(20)	(20)	(21)	(22)	(23)	(23)	(24)	(25)	(25)	(26)	(27)	(27)	(27)	(27)	(30)	(34)	(35)	(35)

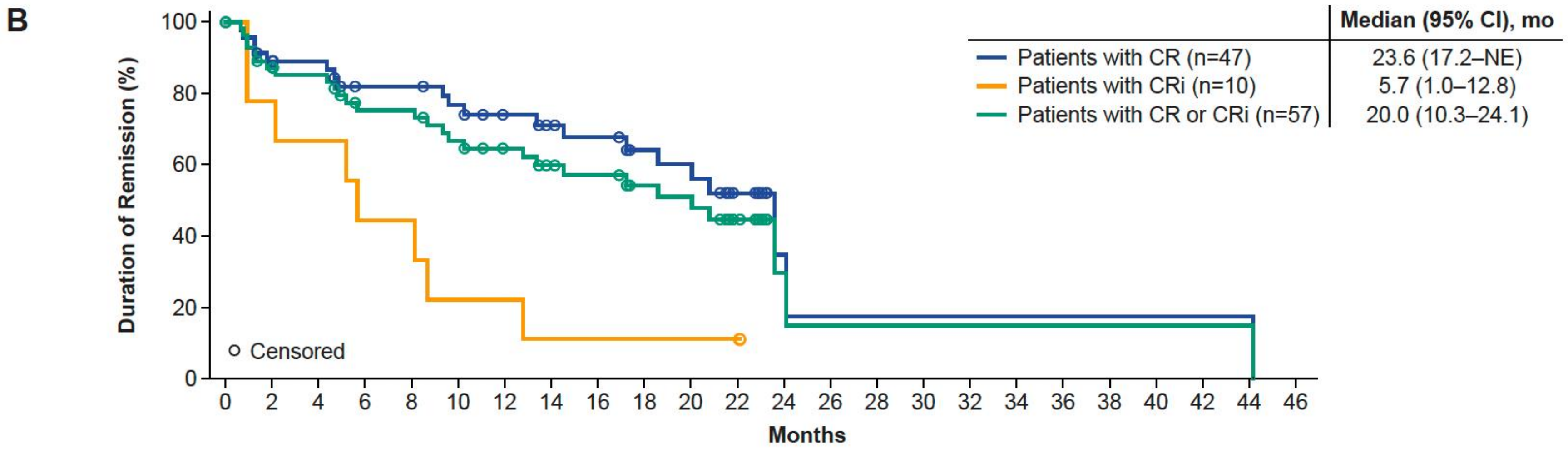

Months	0	2	4	6	8	10	12	14	16	18	20	22	24	26	28	30	32	34	36	38	40	42	44
CR at risk	47	40	38	32	32	29	25	22	20	16	15	9	2	1	1	1	1	1	1	1	1	1	1
(CR censored)	(0)	(2)	(4)	(7)	(7)	(8)	(11)	(13)	(14)	(17)	(17)	(21)	(27)	(27)	(27)	(27)	(27)	(27)	(27)	(27)	(27)	(27)	(27)
CRi at risk	10	7	6	4	4	2	2	1	1	1	1	1	0	0	0	0	0	0	0	0	0	0	0
(CRi censored)	(0)	(1)	(1)	(1)	(1)	(1)	(1)	(1)	(1)	(1)	(1)	(1)	(2)	(2)	(2)	(2)	(2)	(2)	(2)	(2)	(2)	(2)	(2)
CR+CRi at risk	57	47	44	36	36	31	27	23	21	17	16	10	2	1	1	1	1	1	1	1	1	1	1
(CR+CRi censored)	(0)	(3)	(5)	(8)	(8)	(9)	(12)	(14)	(15)	(18)	(18)	(22)	(29)	(29)	(29)	(29)	(29)	(29)	(29)	(29)	(29)	(29)	(29)

C

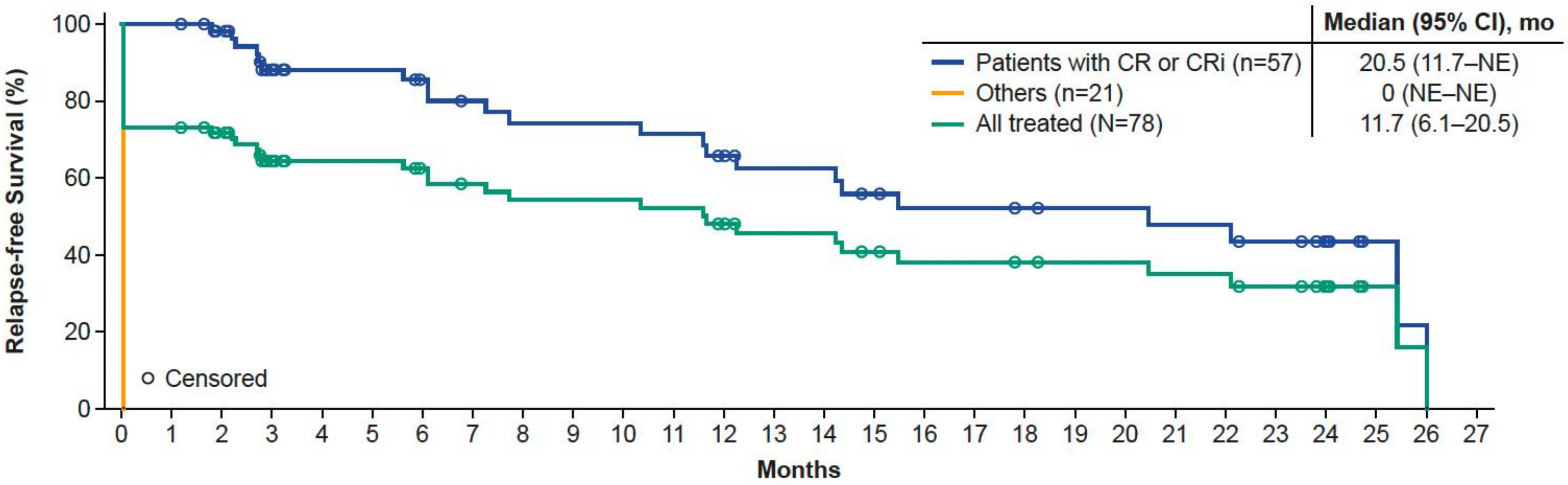

	0	1	2	3	4	5	6	7	8	9	10	11	12	13	14	15	16	17	18	19	20	21	22	23	24	25	26	27
CR+CRi at risk	57	57	51	39	34	34	31	28	26	26	26	25	22	19	19	16	14	14	13	12	12	11	11	9	6	2	1	0
(CR+CRi censored)	(0)	(0)	(5)	(12)	(17)	(17)	(19)	(20)	(20)	(20)	(20)	(20)	(21)	(23)	(23)	(24)	(25)	(25)	(26)	(27)	(27)	(27)	(27)	(28)	(31)	(35)	(35)	(35)
Others at risk	21	0	0	0	0	0	0	0	0	0	0	0	0	0	0	0	0	0	0	0	0	0	0	0	0	0	0	0
(Others censored)	(0)	(0)	(0)	(0)	(0)	(0)	(0)	(0)	(0)	(0)	(0)	(0)	(0)	(0)	(0)	(0)	(0)	(0)	(0)	(0)	(0)	(0)	(0)	(0)	(0)	(0)	(0)	(0)
All Dosed at risk	78	57	51	39	34	34	31	28	26	26	26	25	22	19	19	16	14	14	13	12	12	11	11	9	6	2	1	0
(All Dosed censored)	(0)	(0)	(5)	(12)	(17)	(17)	(19)	(20)	(20)	(20)	(20)	(20)	(21)	(23)	(23)	(24)	(25)	(25)	(26)	(27)	(27)	(27)	(27)	(28)	(31)	(35)	(35)	(35)

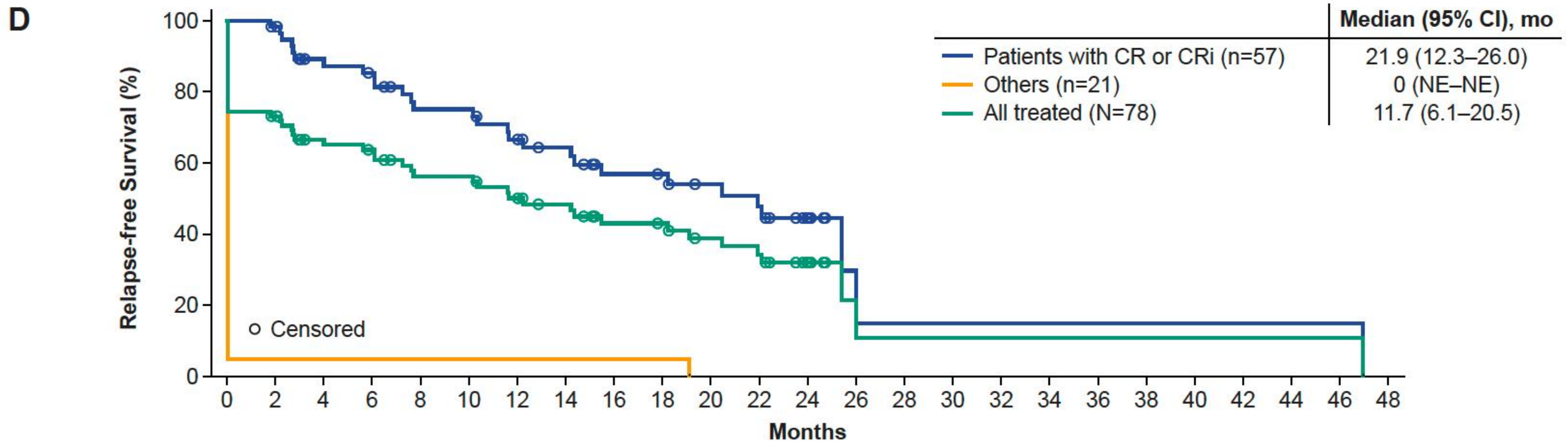

	0	2	4	6	8	10	12	14	16	18	20	22	24	26	28	30	32	34	36	38	40	42	44	46	48
CR+CRi at risk	57	55	46	43	36	36	31	27	21	20	17	15	9	2	1	1	1	1	1	1	1	1	1	1	0
(CR+CRi censored)	(0)	(1)	(5)	(6)	(8)	(8)	(9)	(12)	(15)	(16)	(18)	(18)	(23)	(29)	(29)	(29)	(29)	(29)	(29)	(29)	(29)	(29)	(29)	(29)	(29)
Others at risk	21	1	1	1	1	1	1	1	1	1	0	0	0	0	0	0	0	0	0	0	0	0	0	0	0
(Others censored)	(0)	(0)	(0)	(0)	(0)	(0)	(0)	(0)	(0)	(0)	(0)	(0)	(0)	(0)	(0)	(0)	(0)	(0)	(0)	(0)	(0)	(0)	(0)	(0)	(0)
All Dosedat risk	78	56	47	44	37	37	32	28	22	21	17	15	9	2	1	1	1	1	1	1	1	1	1	1	0
(All Dosed censored)	(0)	(1)	(5)	(6)	(8)	(8)	(9)	(12)	(15)	(16)	(18)	(18)	(23)	(29)	(29)	(29)	(29)	(29)	(29)	(29)	(29)	(29)	(29)	(29)	(29)

E

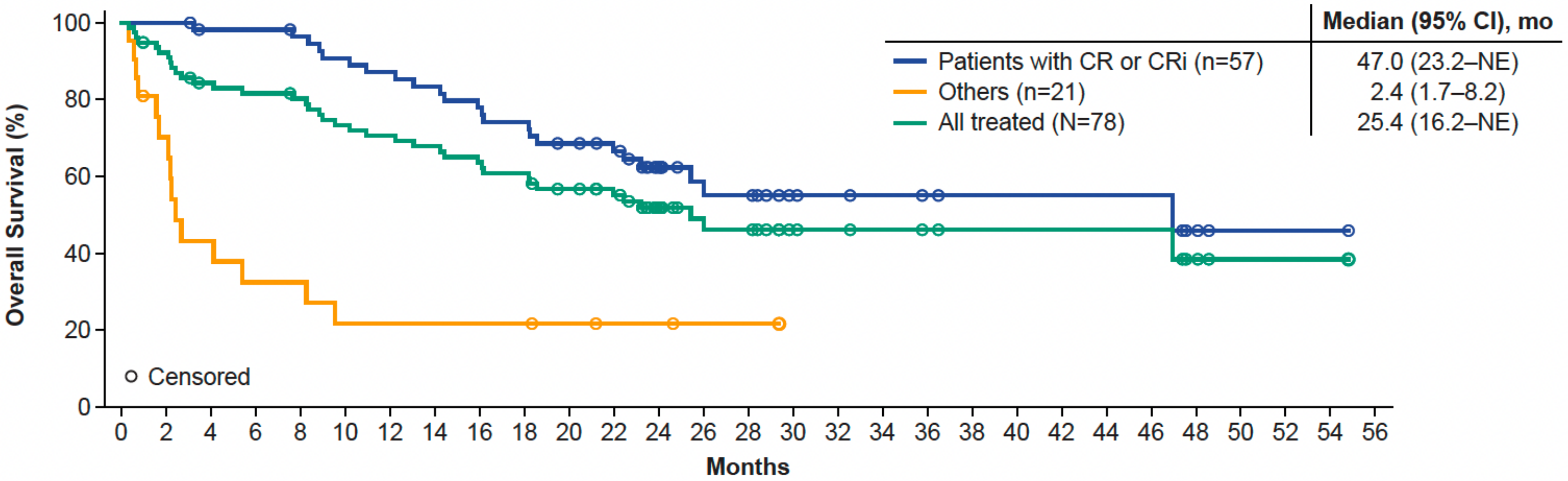

	0	2	4	6	8	10	12	14	16	18	20	22	24	26
CR+CRi at risk	57	57	54	54	52	49	47	45	42	40	36	33	23	16
(CR+CRi censored)	(0)	(0)	(2)	(2)	(3)	(3)	(3)	(3)	(3)	(3)	(4)	(6)	(14)	(20)
Others at risk	21	13	8	6	6	4	4	4	4	4	3	2	2	1
(Others censored)	(0)	(2)	(2)	(2)	(2)	(2)	(2)	(2)	(2)	(2)	(3)	(4)	(4)	(5)
All Dosed at risk	78	70	62	60	58	53	51	49	46	44	39	35	25	17
(All Dosed censored)	(0)	(2)	(4)	(4)	(5)	(5)	(5)	(5)	(5)	(5)	(7)	(10)	(18)	(25)

	28	30	32	34	36	38	40	42	44	46	48	50	52	54	56
CR+CRi at risk	15	10	9	8	7	6	6	6	6	6	3	1	1	1	0
(CR+CRi censored)	(20)	(25)	(26)	(27)	(28)	(29)	(29)	(29)	(29)	(29)	(31)	(33)	(33)	(33)	(34)
Others at risk	1	0	0	0	0	0	0	0	0	0	0	0	0	0	0
(Others censored)	(5)	(6)	(6)	(6)	(6)	(6)	(6)	(6)	(6)	(6)	(6)	(6)	(6)	(6)	(6)
All Dosed at risk	16	10	9	8	7	6	6	6	6	6	3	1	1	1	0
(All Dosed censored)	(25)	(31)	(32)	(33)	(34)	(35)	(35)	(35)	(35)	(35)	(37)	(39)	(39)	(39)	(40)

Figure S5. Kaplan-Meier estimates of OS by age subgroups (18–25, 18–39, 40–59, ≥60 years) in phase 2 treated patients (N=55).

CI=confidence interval. NE=not estimable. NR=not reached. OS=overall survival.

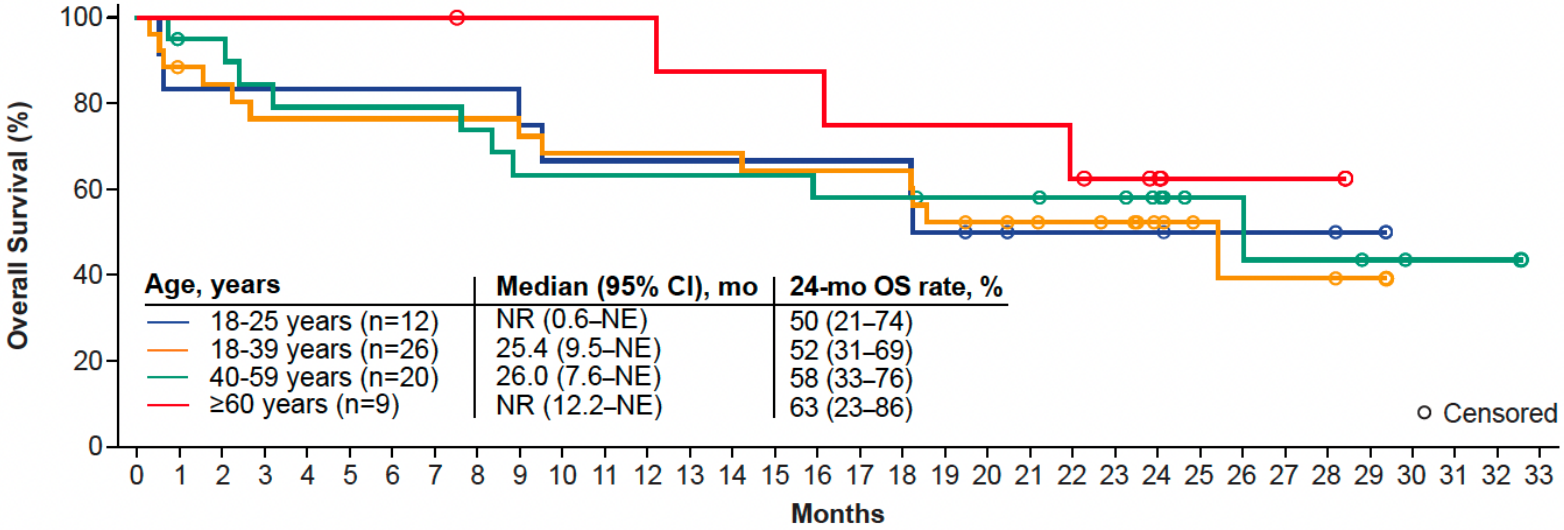

Months	0	1	2	3	4	5	6	7	8	9	10	11	12	13	14	15	16
18-25 at risk	12	10	10	10	10	10	10	10	10	9	8	8	8	8	8	8	8
(18-25 censored)	(0)	(0)	(0)	(0)	(0)	(0)	(0)	(0)	(0)	(0)	(0)	(0)	(0)	(0)	(0)	(0)	(0)
18-39 at risk	26	22	21	19	19	19	19	19	19	18	17	17	17	17	17	16	16
(18-39 censored)	(0)	(1)	(1)	(1)	(1)	(1)	(1)	(1)	(1)	(1)	(1)	(1)	(1)	(1)	(1)	(1)	(1)
40-59 at risk	20	18	18	16	15	15	15	15	14	12	12	12	12	12	12	12	11
(40-59 censored)	(0)	(1)	(1)	(1)	(1)	(1)	(1)	(1)	(1)	(1)	(1)	(1)	(1)	(1)	(1)	(1)	(1)
≥60 Years at risk	9	9	9	9	9	9	9	9	8	8	8	8	8	7	7	7	7
(≥60 Years censored)	(0)	(0)	(0)	(0)	(0)	(0)	(0)	(0)	(1)	(1)	(1)	(1)	(1)	(1)	(1)	(1)	(1)

Months	17	18	19	20	21	22	23	24	25	26	27	28	29	30	31	32	33
18-25 at risk	8	8	6	5	4	4	4	4	3	3	3	3	2	0	0	0	0
(18-25 censored)	(0)	(0)	(0)	(1)	(2)	(2)	(2)	(2)	(3)	(3)	(3)	(3)	(4)	(6)	(6)	(6)	(6)
18-39 at risk	16	16	13	12	11	10	9	6	4	3	3	3	2	0	0	0	0
(18-39 censored)	(1)	(1)	(1)	(2)	(3)	(4)	(5)	(8)	(10)	(10)	(10)	(10)	(11)	(13)	(13)	(13)	(13)
40-59 at risk	11	11	10	10	10	9	9	7	4	4	3	3	2	1	1	1	0
(40-59 censored)	(1)	(1)	(2)	(2)	(2)	(3)	(3)	(5)	(8)	(8)	(8)	(8)	(9)	(10)	(10)	(10)	(11)
≥60 Years at risk	6	6	6	6	6	5	4	3	1	1	1	1	0	0	0	0	0
(≥60 Years censored)	(1)	(1)	(1)	(1)	(1)	(1)	(2)	(3)	(5)	(5)	(5)	(5)	(6)	(6)	(6)	(6)	(6)

Figure S6. Kaplan-Meier estimates of OS by pre-KTE-X19 infusion bone marrow blast percentage subgroups (≤5%, >5%–25%, >25%–50%, >50%–75%, and >75%–100%) in phase 2 treated patients (N=55).

NE=not estimable. NR=not reached. OS=overall survival.

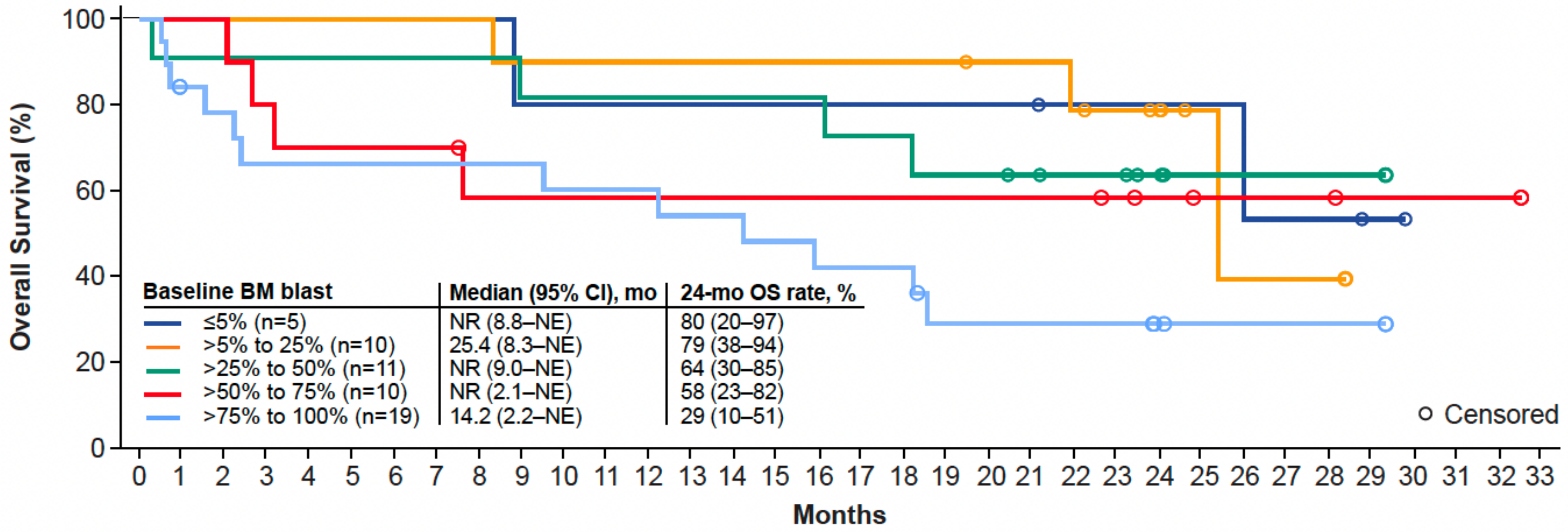

Months	0	1	2	3	4	5	6	7	8	9	10	11	12	13	14	15	16
≤5% at risk	5	5	5	5	5	5	5	5	5	4	4	4	4	4	4	4	4
≤5% censored)	(0)	(0)	(0)	(0)	(0)	(0)	(0)	(0)	(0)	(0)	(0)	(0)	(0)	(0)	(0)	(0)	(0)
>5 to ≤25% at risk	10	10	10	10	10	10	10	10	10	9	9	9	9	9	9	9	9
(>5 to ≤25% censored)	(0)	(0)	(0)	(0)	(0)	(0)	(0)	(0)	(0)	(0)	(0)	(0)	(0)	(0)	(0)	(0)	(0)
>25 to ≤50% at risk	11	10	10	10	10	10	10	10	10	9	9	9	9	9	9	9	9
>25 to ≤50% censored)	(0)	(0)	(0)	(0)	(0)	(0)	(0)	(0)	(0)	(0)	(0)	(0)	(0)	(0)	(0)	(0)	(0)
>50 to ≤75% at risk	10	10	10	8	7	7	7	7	5	5	5	5	5	5	5	5	5
(>50 to ≤75% censored	(0)	(0)	(0)	(0)	(0)	(0)	(0)	(0)	(1)	(1)	(1)	(1)	(1)	(1)	(1)	(1)	(1)
>75 to ≤100% at risk	19	14	13	11	11	11	11	11	11	11	10	10	10	9	9	8	7
(>75 to ≤100% censored)	(0)	(2)	(2)	(2)	(2)	(2)	(2)	(2)	(2)	(2)	(2)	(2)	(2)	(2)	(2)	(2)	(2)

Months	17	18	19	20	21	22	23	24	25	26	27	28	29	30	31	32	33
≤5% at risk	4	4	4	4	4	3	3	3	3	3	2	2	1	0	0	0	0
≤5% censored)	(0)	(0)	(0)	(0)	(0)	(1)	(1)	(1)	(1)	(1)	(1)	(1)	(2)	(3)	(3)	(3)	(3)
>5 to ≤25% at risk	9	9	9	8	8	7	6	5	2	1	1	1	0	0	0	0	0
(>5 to ≤25% censored)	(0)	(0)	(0)	(1)	(1)	(1)	(2)	(3)	(6)	(6)	(6)	(6)	(7)	(7)	(7)	(7)	(7)
>25 to ≤50% at risk	8	8	7	7	6	5	5	3	1	1	1	1	1	0	0	0	0
>25 to ≤50% censored)	(0)	(0)	(0)	(0)	(1)	(2)	(2)	(4)	(6)	(6)	(6)	(6)	(6)	(7)	(7)	(7)	(7)
>50 to ≤75% at risk	5	5	5	5	5	5	4	3	2	2	2	2	1	1	1	1	0
(>50 to ≤75% censored	(1)	(1)	(1)	(1)	(1)	(1)	(2)	(3)	(4)	(4)	(4)	(4)	(5)	(5)	(5)	(5)	(6)
>75 to ≤100% at risk	7	7	4	4	4	4	4	2	1	1	1	1	1	0	0	0	0
(>75 to ≤100% censored)	(2)	(2)	(3)	(3)	(3)	(3)	(3)	(5)	(6)	(6)	(6)	(6)	(6)	(7)	(7)	(7)	(7)

Figure S7. Treatment summary in SCHOLAR-3 cohorts

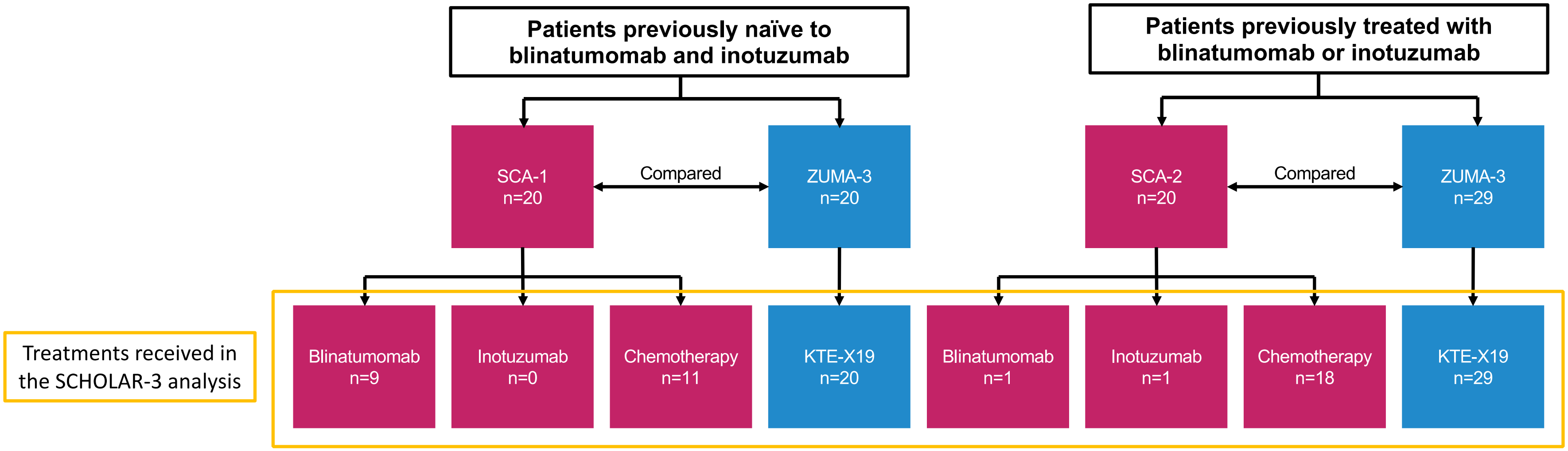

Figure S8. Comparison of CR/CRi rates between matched blinatumomab and inotuzumab-naïve ZUMA-3 patients (n=20) and SCA-1 patients (n=20) by key subgroups.

CR=complete remission. CRi=CR with incomplete hematological recovery. NE=not estimable. SCA=synthetic control arm. SCT=stem cell transplant

Subgroup	N (%) of Patients	ZUMA-3 n/N (%)	SCA-1 n/N (%)	Rate Difference (95% CI)
**Overall**	40 (100)	17/20 (85)	7/20 (35)	50.0 (17.9–73.7)
**Age at baseline**				
< 65 years	34 (85)	14/17 (82)	7/17 (41)	41.2 (7.9–68.5)
≥ 65 years	6 (15)	3/3 (100)	0/3 (0)	100.0 (8.1–100)
**Philadelphia chromosome status**				
Yes	7 (18)	3/4 (75)	2/3 (67)	8.3 (-64.2–75.5)
No	33 (83)	14/16 (88)	5/17 (29)	58.1 (23.0–82.1)
**Percentage bone marrow blasts**				
< 50%	21 (53)	8/9 (89)	5/12 (42)	47.2 (2.7–78.8)
≥ 50%	19 (48)	9/11 (82)	2/8 (25)	56.8 (9.2–86.5)
**Primary refractory status**				
Yes	13 (33)	6/7 (86)	3/6 (50)	35.7 (-19.7–78.8)
No	24 (60)	11/13 (85)	2/11 (18)	66.4 (24.4–89.8)
Unknown	3 (8)	0/0 (NE)	2/3 (67)	NE
**Prior allogeneic SCT**				
Yes	14 (35)	5/7 (71)	2/7 (29)	42.9 (-16.2–83.2)
No	26 (65)	12/13 (92)	5/13 (39)	53.8 (17.8–80.9)
**1st relapse with 1st remission ≤ 12 months**				
Yes	11 (28)	7/8 (88)	0/3 (0)	87.5 (15.8–99.7)
No	26 (65)	10/12 (83)	5/14 (36)	47.6 (8.7–76.7)
Unknown	3 (8)	0/0 (NE)	2/3 (67)	NE
**No. of lines of prior therapy**				
≤ 2	32 (80)	14/16 (88)	5/16 (31)	56.3 (21.9–80.7)
> 2	8 (20)	3/4 (75)	2/4 (50)	25.0 (-51.0–83.0)

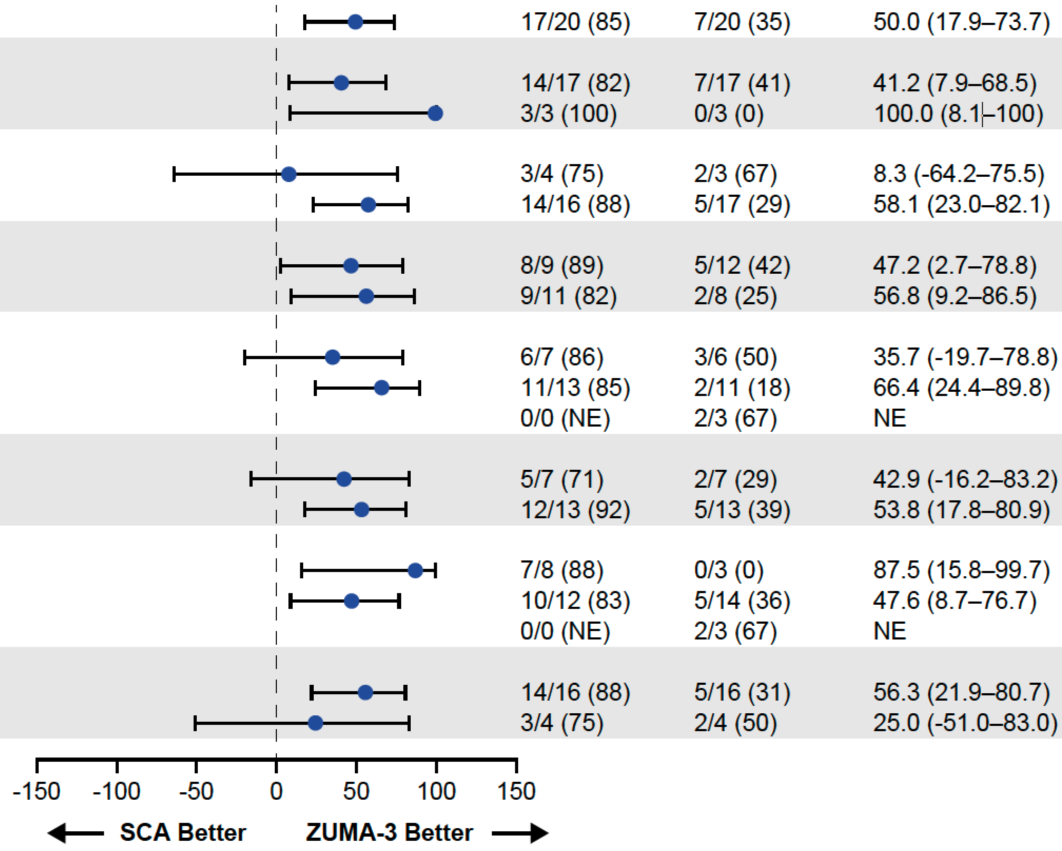

Figure S9. Kaplan-Meier curves of RFS for matched blinatumomab and inotuzumab-naïve ZUMA-3 patients (n=20) and SCA-1 patients (n=20).

RFS=relapse-free survival. SCA=synthetic control arm.

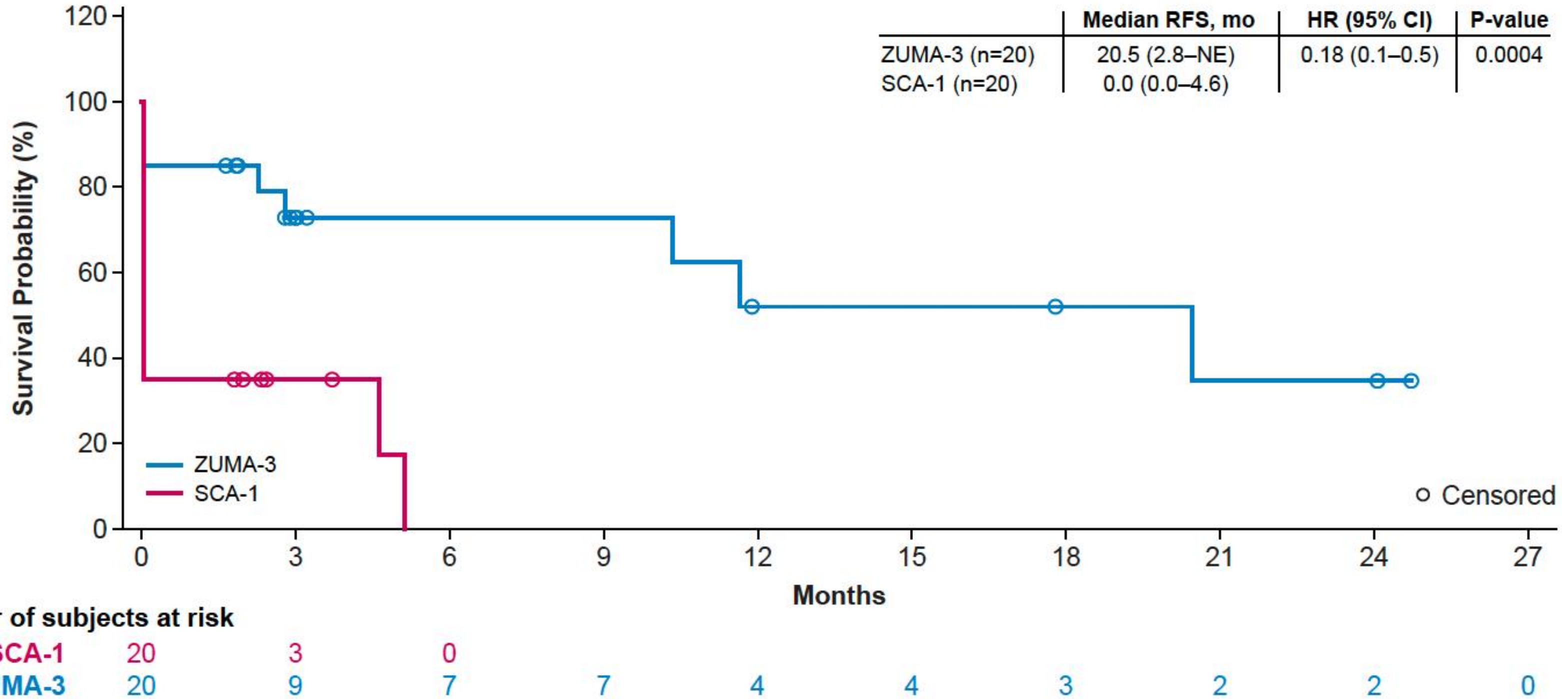

	Median RFS, mo	HR (95% CI)	P-value
ZUMA-3 (n=20)	20.5 (2.8–NE)	0.18 (0.1–0.5)	0.0004
SCA-1 (n=20)	0.0 (0.0–4.6)		

Number of subjects at risk

	0	3	6	9	12	15	18	21	24	27
SCA-1	20	3	0							
ZUMA-3	20	9	7	7	4	4	3	2	2	0

Supplementary References

1. Shah BD, Ghobadi A, Oluwole OO, et al. KTE-X19 for relapsed or refractory adult B-cell acute lymphoblastic leukaemia: phase 2 results of the single-arm, open-label, multicentre ZUMA-3 study. *Lancet* 2021; **398**(10299): 491-502.

2. Shah BD, Bishop MR, Oluwole OO, et al. KTE-X19 anti-CD19 CAR T-cell therapy in adult relapsed/refractory acute lymphoblastic leukemia: ZUMA-3 phase 1 results. *Blood* 2021; **138**(1): 11-22.
